# DEMO2: Assemble multi-domain protein structures by coupling analogous template alignments with deep-learning inter-domain restraint prediction

**DOI:** 10.1093/nar/gkac340

**Published:** 2022-05-10

**Authors:** Xiaogen Zhou, Chunxiang Peng, Wei Zheng, Yang Li, Guijun Zhang, Yang Zhang

**Affiliations:** Department of Computational Medicine and Bioinformatics, University of Michigan, Ann Arbor, MI 48109, USA; College of Information Engineering, Zhejiang University of Technology, Hangzhou 310023, China; College of Information Engineering, Zhejiang University of Technology, Hangzhou 310023, China; Department of Computational Medicine and Bioinformatics, University of Michigan, Ann Arbor, MI 48109, USA; Department of Computational Medicine and Bioinformatics, University of Michigan, Ann Arbor, MI 48109, USA; College of Information Engineering, Zhejiang University of Technology, Hangzhou 310023, China; Department of Computational Medicine and Bioinformatics, University of Michigan, Ann Arbor, MI 48109, USA; Department of Biological Chemistry, University of Michigan, Ann Arbor, MI 48109, USA

## Abstract

Most proteins in nature contain multiple folding units (or domains). The revolutionary success of AlphaFold2 in single-domain structure prediction showed potential to extend deep-learning techniques for multi-domain structure modeling. This work presents a significantly improved method, DEMO2, which integrates analogous template structural alignments with deep-learning techniques for high-accuracy domain structure assembly. Starting from individual domain models, inter-domain spatial restraints are first predicted with deep residual convolutional networks, where full-length structure models are assembled using L-BFGS simulations under the guidance of a hybrid energy function combining deep-learning restraints and analogous multi-domain template alignments searched from the PDB. The output of DEMO2 contains deep-learning inter-domain restraints, top-ranked multi-domain structure templates, and up to five full-length structure models. DEMO2 was tested on a large-scale benchmark and the blind CASP14 experiment, where DEMO2 was shown to significantly outperform its predecessor and the state-of-the-art protein structure prediction methods. By integrating with new deep-learning techniques, DEMO2 should help fill the rapidly increasing gap between the improved ability of tertiary structure determination and the high demand for the high-quality multi-domain protein structures. The DEMO2 server is available at https://zhanggroup.org/DEMO/.

## Graphical Abstract

**Graphical Abstract:**
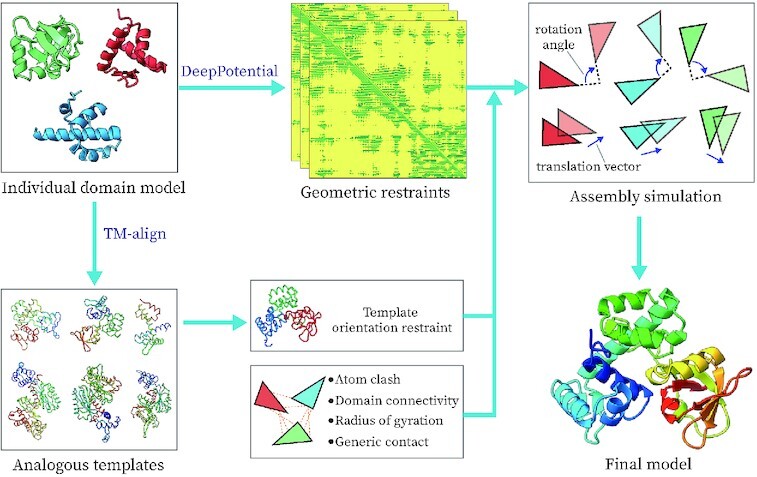
DEMO2 is an significantly improved version for automated assembly of full-length structural models of multi-domain proteins by integrating analogous template alignments with deep-learning predicted inter-domain spatial restraints.

## INTRODUCTION

Deep-learning techniques have dramatically promoted the progress of protein structure prediction (1–4), especially the end-to-end sequence-to-structure AlphaFold2 (5) has achieved unprecedented modeling accuracy in the protein structure prediction (6,7). Based on the analysis of AlphaFold2 models for all human proteins, the confidence score of the AlphaFold2 model is highly correlated with whether the target has homologs in the Protein Data Bank (PDB) or not (8). However, many proteins have no homologous full-length templates, especially for proteins containing multiple folding units (called domains). In fact, most proteins in nature consist of more than two domains (9). Due to the much higher degree of freedoms in domain-orientation space and the stability of full-length structures often involving interactions with other protein cofactors, the majority of the multi-domain proteins have been solved as single-domain proteins, and only 35.3% of proteins in the PDB contain multiple domains (10). As a result, many methods, including the end-to-end deep-learning approaches, can correctly build the domain models but cannot accurately predict the domain orientations (6).

We previously developed a pipeline, DEMO (11), for constructing multi-domain protein structures by docking-based domain assembly simulations, with inter-domain orientations determined by the distance profiles from analogous templates as detected through domain-level structure alignments. Taking advantage of the complementary information deduced from the structural analogous templates and the physics-based steric potentials, DEMO outperformed other domain assembly programs and structure modeling methods, especially for the cases without homologous full-length templates (11,12). Due to the robustness of the results and user-friendly server design, DEMO server (since September, 2019) has assembled structures for > 3,000 proteins submitted by > 1,000 users from 42 countries. Given the rapid development of deep learning techniques in the field, however, the previous DEMO server may no longer represent the state-of-the-art, and the revolutionary success of AlphaFold2 in single-domain structure prediction showed potential to extend deep-learning techniques for multi-domain structure modeling. In addition, the DEMO simulation was built on iterative assembly of pair-wise consecutive domains, which can often be biased to the order of domain assembly, without detecting the optimal domain orientations for proteins with three or more domains. Therefore, an upgraded version of the server integrating deep learning technology and with the ability to simultaneously optimize the orientations of all domain models is urgently needed. It should be noted that although AlphaFold2 could generate multi-domain models from query sequence with reasonable accuracy (5), it is not designed to assemble full-length models directly from domain structures, which is a long-time problem faced by many researchers in the community.

In this work, we developed a significantly improved method, DEMO2, which integrates analogous template alignments with deep-learning techniques for high-accuracy domain structure assembly. Starting from individual domain models, inter-domain spatial restraints are first predicted with deep residual convolutional networks, where full-length structure models are constructed by simultaneously searching for the optimal positions and orientations of all domains using Limited-memory Broyden-Fletcher-Goldfarb-Shanno (L-BFGS) simulations, under the guidance of a hybrid energy function combining deep-learning restraints and analogous template alignments. The results on the large-scale benchmark and CASP blind tests showed that DEMO2 significantly improves the performance compared to its predecessor and outperforms the best linker-based domain assembly methods and the state-of-the-art structure modeling tools.

## MATERIAL AND METHODS

### Pipeline of DEMO2 server

**Figure 1.**
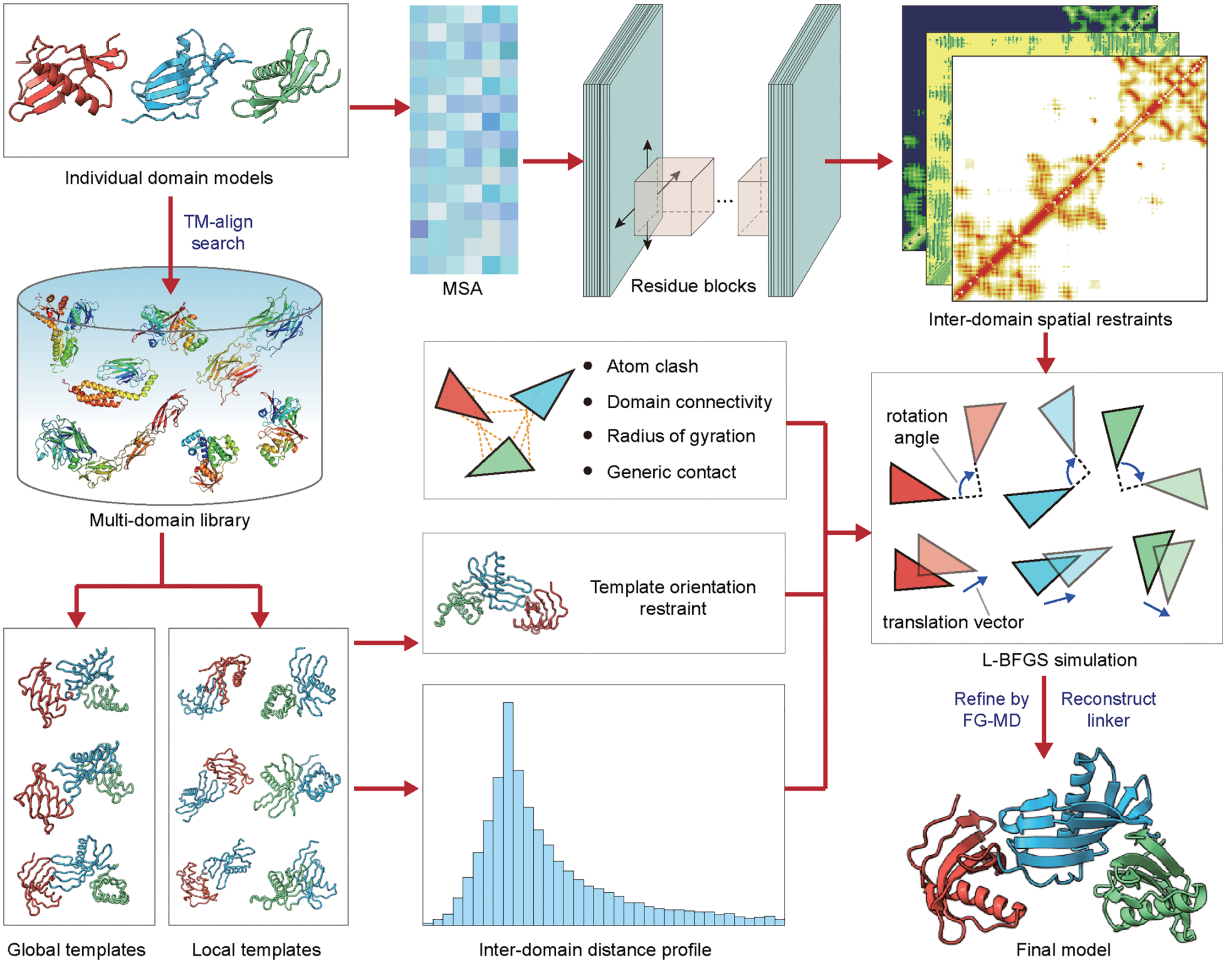
Flowchart of the DEMO2 pipeline. The procedure mainly includes global and local templates identification, inter-domain spatial restraints prediction by DeepPotential, domain model assembly through fast L-BFGS simulation, and side-chain repacking and domain-domain linker reconstruction.

DEMO2 is a pipeline for automated assembly of full-length structural models of multi-domain proteins by integrating analogous template structural alignments with deep-learning predicted inter-domain spatial restraints. As shown in Figure 1, starting from individual domain sequences and structures, multiple sequence alignments (MSA) are first constructed by iteratively searching the query against the meta-genome sequence database and two whole-genome databases using DeepMSA (13). Here, the MSA is constructed based on the full-chain sequence merged according to the domain order or provided by users, rather than searching/combining the MSA of each domain independently. Next, the full-chain MSAs are fed into a deep residual convolutional neural network, DeepPotential (14), to predict the inter-domain spatial restraints, including residue-residue distances, torsion angles, and hydrogen-bonding networks. Meanwhile, the global and local multi-domain structure templates, which have similar component domains to the query, are identified by structurally threading the input domains through a non-redundant multi-domain structural library. Full-length models are then assembled by a fast L-BFGS based rigid-body domain structure assembly simulation, which is guided by a hybrid energy function consisting of inter-domain deep-learning restraints, inter-domain distance profiles collected from the top-ranked analogous full-length templates, and physics-based steric potentials (as well as the cross-linking and cryo-EM density map data if available). The L-BFGS assembled models are further submitted for linker reconstruction followed by atomic-level refinement with fragment-guided molecule dynamics (FG-MD) simulations (15), with the low-energy conformations returned as final models.

### New developments in DEMO2

Compared to the previous DEMO (11), four major developments were introduced to DEMO2, including global template identification, deep-learning inter-domain geometric restraints, fast L-BFGS simulation for domain orientation modeling, and cryo-EM data guided domain rigid-body assembly and flexible refinement for high accurate modeling. In the following, we briefly describe these improvements.

#### Global templates identification

In addition to the local templates for the pairwise consecutive domains employed in DEMO, global templates that cover as many domains as possible are introduced to DEMO2. The global templates are identified from the multi-domain protein structure library through a local evaluation followed by a global evaluation based on the analogous structural alignments (Supplementary Figure S1A). For each template in the library, the local evaluation (Supplementary Figure S1B) is firstly performed by structurally aligning each domain model to the template through TM-align (16), where the overlap between the alignments of different domains is allowed. The harmonic mean of the TM-scores of all domains is defined as the score of the template:

(1) $$\begin{equation*}{\rm{TM}} - {\rm{scor}}{{\rm{e}}_{\rm{h}}} = \frac{{{N_{{\rm{dom}}}}}}{{\mathop \sum \nolimits_{d = 1}^{{N_{{\rm{dom}}}}} \frac{1}{{{\rm{TM}} - {\rm{scor}}{{\rm{e}}_d}}}}}\ \end{equation*}$$

where ${N_{{\rm{dom}}}}$ is the total number of domains to be assembled; ${\rm{TM}} - {\rm{scor}}{{\rm{e}}_d}$ is the TM-score between the *d*th domain model and the template structure. According to the TM-score_h_, the top 500 templates are then selected to perform the global evaluation, where the overlap between the alignments of different domains is not allowed (Supplementary Figure S1C). The domains in the global evaluation are aligned from the N to C terminal and from C to N terminal, respectively, and the alignment with the higher TM-score_h_ is considered as the global template. If the best global template with the highest TM-score_h_ cannot cover all domains (e.g. one of the domains has the TM-score < 0.5), the templates of the two broken parts will be independently detected (Supplementary Figure S1D). Meanwhile, the local templates for every two consecutive domains are also obtained if the target contains $ \ge$3 domains. Finally, the top 10 global templates and local templates are selected respectively to build multiple initial full-length models according to a sliding-window based procedure since the aligned regions of the domains separately detected by TM-align may be far away from each other. Details of the procedure for initial conformation construction can be found in Supplementary Text S1 and Supplementary Figure S2.

#### Incorporating deep-learning restraints

Four deep-learning inter-domain geometric restraints predicted by DeepPotential (14) are introduced into DEMO2, including inter-domain distances, domain-domain interface contacts, inter-domain orientations, and hydrogen-bond networks (17). Here, the domain-domain interface contact maps are generated by summing the cumulative probability of $C\alpha$ distances < 18 Å in the predicted distances maps. These four restraints are incorporated with the DEMO inherent energy items including local domain orientation restraints from the initial domain-template superposition, inter-domain distance restraints deduced from the top templates, inter-domain steric clashes, a generic inter-domain contact potential, and a domain boundary connectivity restraint, to build a hybrid energy function to guide the assembly. The full energy function is described in detail in Supplementary Text S2.

#### L-BFGS simulation for domain assembly

Instead of iteratively assembling every two consecutive domains through the replica-exchange Monte Carlo (REMC) simulations in DEMO, DEMO2 performs an L-BFGS simulation to simultaneously assemble all domain structures. Starting from the initial full-length model built from the template, the full-length structure decoy is created according to the corresponding inter-domain rotation angles and translation vectors, as sampled by the L-BFGS simulation for each domain (Supplementary Text S3), which is guided by the hybrid DEMO2 energy function. For each of the ten initial models generated by the top global templates and ten initial models created by the top local templates, the L-BFGS simulation is independently performed with 200 steps, and the final full-length model is built according to the translation vector and rotation angles with the lowest energy (Supplementary Text S3).

#### Cryo-EM data assisted domain rigid-body and flexible assembly

When the cryo-EM data is available for the target, DEMO2 employs a new strategy to process the rigid-body domain assembly simulation. Instead of fitting the initial full-length model created by the template into the density map in DEMO, DEMO2 creates the initial model by matching each domain model into the density map based on the best position and orientation identified by L-BFGS, where the L-BFGS simulation is purely guided by the density correlation (11) between the domain model and the density map. Starting from multiple initial models generated by combining the top poses of each domain, a second L-BFGS simulation is performed to optimize the orientations of each domain under the guidance of the inherent DEMO2 energy and the cryo-EM density correlation score. According to the density correlation score between the full-length model and the density map, the top models from the rigid-body L-BFGS assembly are selected to perform the atom-, segment-, and domain-level refinements using REMC simulations, under the guidance of an atomic-level force field combining the QUARK knowledge-based force field (2), DeepPotential predicted inter-domain restraints, and the density correlation score. Finally, the lowest-energy decoy is selected to construct the final model, with the side-chain atoms repacked by FASPR (18) followed by the FG-MD (15) refinements.

#### Model quality estimation

In order to facilitate the interpretation of the predicted models, the estimated TM-score (eTM-score) and RMSD (eRMSD) are introduced to DEMO2 to estimate the accuracy of the assembled model. They are calculated according to the significance of the templates identified by analogous structural alignments using all domain models, the consistency between the deep-learning predicted inter-domain distances/contacts and those in the assembled model, the convergence of the domain model assembly simulations, and the estimated accuracy of the individual domain models by ResQ (19). Details for the eTM-score and eRMSD calculation can be found in Supplementary Text S4. The eTM-score and eRMSD was tested over the DEMO benchmark set of 356 non-redundant multi-domain proteins with different domain types. The Pearson correlation coefficient (PCC) between eTM-score and the actual TM-score is 0.85 (Supplementary Figure S3A), corresponding to an average error of 0.07. The PCC between eRMSD and actual RMSD is 0.82 (Supplementary Figure S3B), where the average error is 2.2Å. When an eTM-score cutoff of 0.5 is used to select models with correct global topologies (20), both the false-negative and false-positive rates are < 0.15, indicating the quality prediction by eTM-score is correct for > 85% cases. It should be noted that RMSD is sensitive to the local variations as it uses the same weight for all residue pairs in the calculation (Supplementary Text S5), which results in the improper evaluation for the global topology (21). In this regard, we recommend using TM-score as a more reliable measurement for the model accuracy assessment.

## RESULTS

### Comparison with other domain-structure assembly methods

To evaluate DEMO2, we tested it on a comprehensive benchmark set which is extended from the original DEMO benchmark set (11) and with a sequence identity < 30% to the proteins in the training set of DEMO2. Meanwhile, all proteins overlapped with the DeepPotential training dataset have been excluded. This benchmark set contains 461 proteins (Supplementary Table S1) with 2 to 10 domains, including 260 proteins with continuous domains and 201 proteins with $ \ge 1$ discontinuous domains (see examples shown in Supplementary Figure S4). The structures of individual domain models were modelled by D-I-TASSER (12), where all homologous templates with a sequence identity > 30% to the query have been excluded; this resulted in the TM-score of domain models ranging from 0.17 to 0.97 with an average TM-score = 0.77.

We first compare DEMO2 with its predecessor DEMO (11) and the linker-based domain assembly method AIDA (22). Table 1 summarizes the results of full-length models assembled by AIDA, DEMO, and DEMO2, using the same domain models. On average, DEMO2 models obtain a higher TM-score and a lower RMSD than models constructed by the control methods for all categories of domain structures (Supplementary Table S1). Overall, DEMO2 models achieve an average TM-score of 0.71, which is 36.5% and 12.7% higher than that built by AIDA (0.52) and DEMO (0.63), respectively. The Student's t-test P-values are 3.02E-51 and 1.77E-09 for DEMO2 vs. AIDA and DEMO2 vs. DEMO, respectively, indicating statistically significant differences between the methods. In addition, as a more stringent examination of inter-domain orientations, DEMO2 models achieve a much higher rTM-score (0.48) than either AIDA (0.23) or DEMO (0.38). Here, rTM-score is a metric to evaluate the domain orientation and calculated according to the TM-score with only one-time alignment for the two models (see Eq. S16 in Supplementary Text S5).

**Table 1.** Results of full-length models built by different methods over 461 test multi-domain proteins. Bold font highlights the best results. P-value is calculated between DEMO2 and the control methods through two-tailed Student's T-test

	TM-score	rTM-score	RMSD (Å)	P-value
AIDA	0.52	0.23	16.2	3.02E-51
DEMO	0.63	0.38	12.6	1.77E-09
DMPfold	0.57	0.34	14.0	8.11E-23
trRosetta	0.64	0.41	11.2	5.86E-07
DEMO2	**0.71**	**0.48**	**9.4**	-

**Figure 2.**
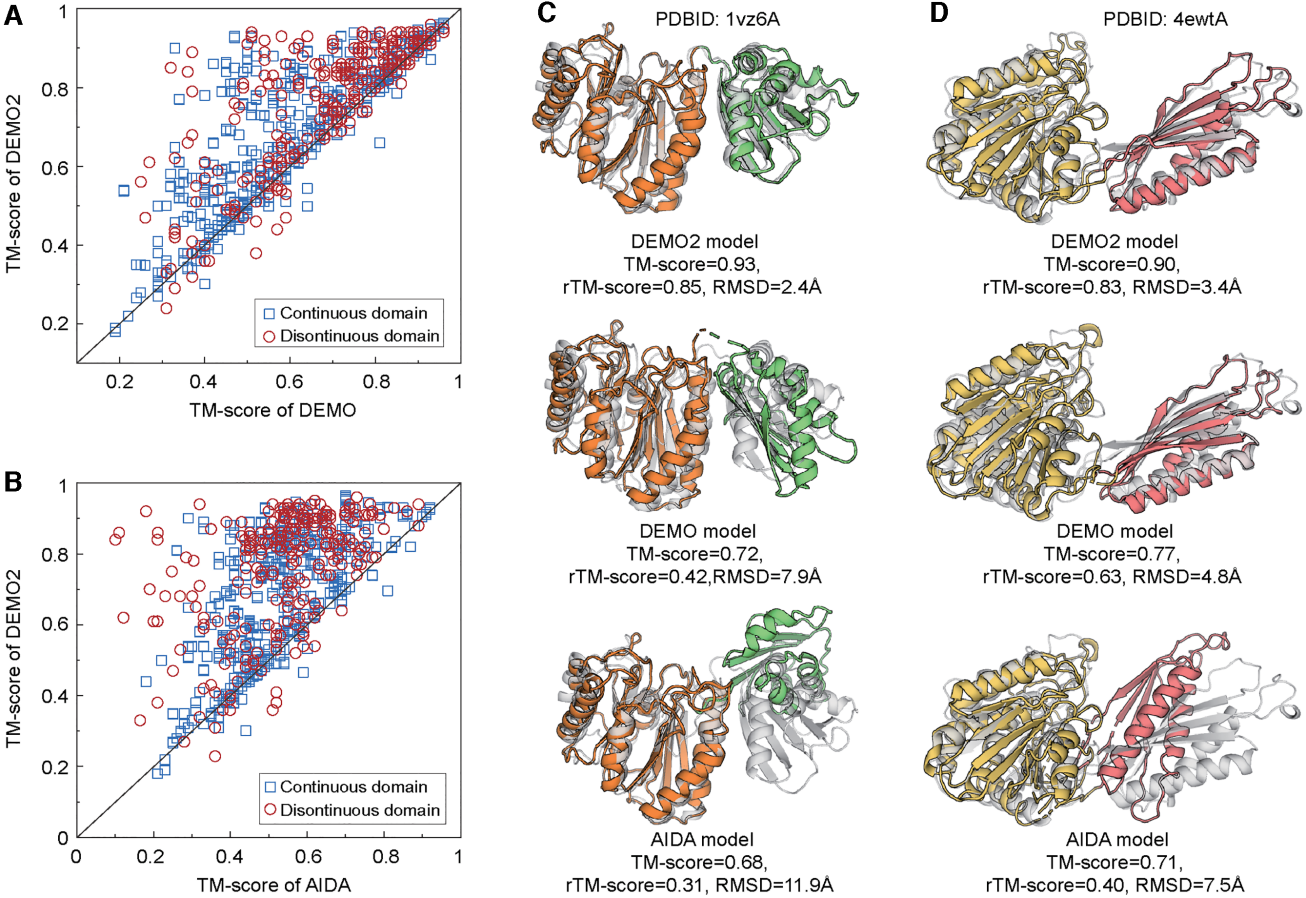
Comparison of DEMO2 with AIDA and DEMO. (**A**) Head-to-head TM-score comparison of models assembled by DEMO2 and that created by DEMO. (**B**) Head-to-head TM-score comparison of models generated by DEMO2 and that built by AIDA. (**C** and **D**) representative examples are showing DEMO2 builds better full-length models than DEMO and AIDA. Gray and color cartoons are native structures and DEMO assembled models, respectively, and different domains in the assembled models are represented by different colors. (C) 1vz6A. (D) 4ewtA.

Figure 2A and B present the head-to-head comparison of DEMO2 versus DEMO and AIDA on the TM-score, respectively. DEMO2 obtains a higher TM-score than DEMO and AIDA in 369 and 413 out of 461 proteins, respectively. Accordingly, DEMO2 models achieve correct global topologies (TM-score > 0.5) (20) on 388 cases, which is 17.2% and 41.6% higher than that of DEMO (331) and AIDA (274), respectively. Interestingly, proteins with discontinuous domains are usually difficult to model as they have several motifs separated in the sequence which increases the difficulty in both individual domain modeling and inter-domain restraints prediction. However, probably because the inserted domains provided additional anchor restraints on domain orientations, DEMO2 assembled models with the correct global fold on 87.6% cases of discontinuous domains, which is higher than that of proteins with continuous domains (79.2%); this rate is 8.6% and 32.3% higher than that of DEMO (80.6%) and AIDA (66.2%), respectively.

Figure 2C and D illustrate examples from *Ornithine Acetyltransferase* (PDBID: 1vz6A), a protein with two continuous domains, and *methicillin resistant Staphylococcus aureus* (PDBID: 4ewtA), a protein that consists of a continuous domain and discontinuous domain, respectively. For 1vz6A (Figure 2C), although D-I-TASSER correctly created all domain models (TM-score = 0.94 and 0.88), the best analogous template detected based on the domains obtained a TM-score = 0.66 to the native structure, which resulted in a final model with TM-score = 0.72 by DEMO as its assembly is mainly based on the templates. However, DEMO2 correctly modelled the domain orientation and obtained a final model with TM-score = 0.93 since DeepPotential correctly predicted the inter-domain distance with an average error of 0.76 Å. Because AIDA is a linker-based modeling method that usually leaves the domain orientations largely random, the final model obtains a TM-score of 0.68; this relative reasonable TM-score for the AIDA is mainly due to the correct domain models from D-I-TASSER, as the rTM-score is 0.31, which is significantly lower than that of the DEMO2 model (0.85). For 4ewtA (Figure 2D), a promising analogous template with TM-score = 0.75 to the native structure was detected from the library. Based on the template, DEMO slightly improved the model and resulted in a final model with TM-score = 0.77. When combining the template with the inter-domain restraints predicted by DeepPotential, the final model was significantly improved with TM-score increased to 0.90 by DEMO2. AIDA also failed to determine the domain orientation, resulting in a final model with TM-score = 0.71. Again, the rTM-score of the DEMO2 model (0.83) is significantly higher than both models from DEMO (0.63) and AIDA (0.40). These results further reinforce the advantage by integrating analogous templates with deep-leaning inter-domain restraints.

### Comparison with deep-learning structure prediction methods

**Figure 3.**
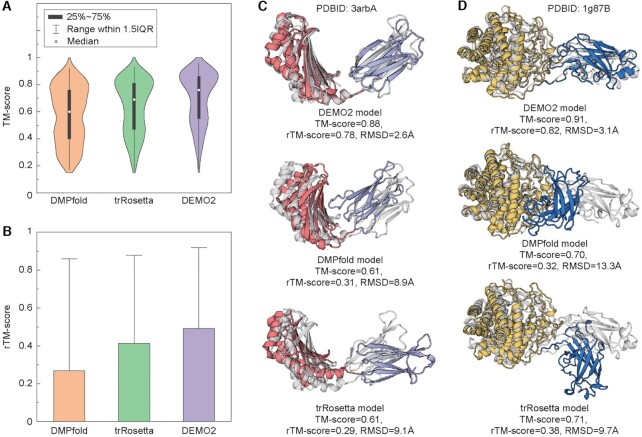
Comparison of DEMO2 with DMPfold and trRosetta. (**A**) Violin plot plus box plot for the TM-score of the final full-length model, where IQR means the interquartile range of the TM-score. (**B**) Histogram of the rTM-score of the final full-length model, where the vertical line indicates the outlier of the TM-scores. (**C** and **D**) representative examples are showing DEMO2 creates more accurate models than DMPfold and trRosetta. Gray and color cartoons are native structures and DEMO2 assembled models, respectively, and different domains in the assembled models are represented by different colors. (C) 3arbA. (D) 1g87B.

In this section, DEMO2 is compared with two widely deep-learning structure modeling tools, trRosetta (23) and DMPfold (24). The results of full-length models directly generated by trRosetta and DMPfold are also summarized in Table 1, Figure 3A and B. The average TM-score of DEMO2 models (0.71) is 24.6% higher than that of DMPfold (0.57) and 10.9% higher than that of trRosetta (0.64); the Student's t-test *P*-value is 8.11E-23 and 5.86E-07, respectively, relative to DMPfold and trRosetta, showing statistical differences. Meanwhile, the average rTM-score is also higher than that of DMPfold and trRosetta, showing better domain orientations modelled by DEMO2 compared with the control methods.

Supplementary Figure S5A and S5B show the head-to-head comparisons of the TM-score of the DEMO2 model versus those generated by DMPfold and trRosetta, respectively. It can be found that DEMO2 obtains a higher TM-score than DMPfold and trRosetta in 440 and 411 proteins, while DMPfold and trRosetta achieves a higher TM-score in only 21 and 50 cases, respectively. As a result, DMPfold and trRosetta models have TM-score > 0.5 on 269 and 326 cases, which is 44.2% and 19.0% lower than that of DEMO2 (388), respectively. In addition, Figure 3A and B also display the TM-score distribution and the rTM-score histogram of different methods, which again show that DEMO2 can predict more accurate domain orientations and thus resulting in better full-length models.

In addition, we also compared DEMO2 with DMPfold and trRosetta on a subset of 162 proteins for which all proteins with sequence identity > 30% to the DeepPotential training set have been excluded. As shown in Supplementary Figure S6, DEMO2 still obtains reasonable models with a better accuracy than DMPfold and trRosetta. On average, DEMO2 achieves a TM-score of 0.70, which is 23.8% and 11.1% higher than that of DMPfold and trRosetta, respectively. The respective *P*-values on Student's t-test are 3.43E-11 and 4.63E-05, indicating statistically significant differences between the methods. DEMO2 models achieve correct global topologies on 136 cases, which is 47.8% and 23.6% higher than that of DMPfold (92) and trRosetta (110), respectively. In the aspect of inter-domain orientations, DEMO2 models also obtain a higher rTM-score (0.48) than either DMPfold (0.33) or trRosetta (0.41).

Given that both DEMO2 and trRosetta/DMPfold simulations utilized the deep-learning restraints, these results reflect the advantage of the DEMO2 protocol which split the proteins into domains and then model the domain structure individually. Since the deep-learning methods are trained mainly on the single-domain proteins (-because most proteins in the PDB are solved as individual domains), this may result in the incorrect folding for some domains when directly modelling full-length structures of the multi-domain proteins. Since the D-I-TASSER deep-learning model is also trained through single-domain proteins, the DEMO2-based domain split allows it to construct better models for individual domains. In Supplementary Figure S7, we compare the individual domain models (1202) from all test proteins by different methods, where the TM-score of the models by D-I-TASSER ( = 0.77), which are used by DEMO2, is 28.3% and 10.0% higher than that of DMPfold (0.60) and trRosetta (0.70), respectively. Interestingly, if we additionally excluded all templates with a TM-score > 0.5 to the target structure in the individual domain modeling, the quality of the D-I-TASSER models is only marginally reduced (TM-score = 0.76), probably due to the utilization of the deep-learning component of D-I-TASSER which is independent upon the quality of PDB templates. Accordingly, when feeding these domain models into the DEMO2, the quality of full-chain model is almost unchanged with the average TM-score ( = 0.70), which is again higher than the full-length models by DMPfold (0.57) and trRosetta (0.64). Additionally, the analogous templates detected by the domain-level structural alignments, which use the D-I-TASSER domain models as probes, can provide additional restraints, which are complementary to the deep-learning inter-domain restraints, to guide the domain assembly simulations of DEMO2.

Figure 3C and D present two representative examples from the *NKT TCR-CD1d-alpha- galactosylceramide analogue-OCH* (PDBID: 3arbA) and the *Endoglucanase 9G* (PDBID: 1g87B). For 3arbA, all methods correctly modelled the individual domain structures with TM-score > 0.8. However, the domain orientations were not correctly predicted by DMPfold and trRosetta as indicated by the rTM-score (0.31 and 0.29), which resulted in the full-length model with a relative low TM-score = 0.61 for both DMPfold and trRosetta (Figure 3C). DEMO2 correctly modelled the domain orientations and obtained a full-length model with rTM-score/TM-score = 0.78/0.88. For 1g87B, DMPfold misfolded the smaller-size domain (TM-score = 0.3) and resulted in the incorrect overall domain orientation with rTM-score = 0.32 and TM-score = 0.70 for the full-length model (Figure 3D). Although trRosetta correctly predicted all domain models (TM-score = 0.9 and 0.76), the inter-domain orientations were not correctly generated, resulting in a full-length model with a poorer rTM-score/TM-score = 0.38/0.71. DEMO2 model obtains an rTM-score/TM-score = 0.82/0.91 since it correctly generates inter-domain orientations based on the accurate domain models (TM-score = 0.92 and 0.87) created by D-I-TASSER, under the guidance of the complementary template and deep-learning restraints. The results further demonstrate the advantage of the DEMO2 protocol for multi-domain protein full-length structures modeling.

### Blind test of DEMO2 on the CASP14 targets

DEMO2 was also used to assemble structures of all multi-domain targets in the most recent community-wide CASP experiment for the Zhang group servers (‘Zhang-Server’ and ‘QUARK’) in fully automated protein structure prediction categories. Here we take Zhang-Server as an example since it utilized the D-I-TASSER algorithm to build the domain level models. Since both DeepPotential and DEMO2 were trained before CASP14, none of the CASP14 targets was included in their training dataset. Supplementary Figure S8 presents a summary of the best five servers in CASP14 after excluding the other Zhang group servers, in which the servers were sorted according to the average TM-score of the full-length models for all multi-domain proteins with at least one template-free modeling (FM) or template-free modeling/template-based modeling (FM/TBM) domains. Since the domain structures of all target proteins are unknown when participating in the CASP, the domain boundaries of all targets were predicted from the sequence by a deep-learning contact-based program FUpred (19) and a threading template-based method ThreaDom (20) in Zhang-Server, which resulted in an average normalized domain overlap (NDO) score (25) of 0.86. On average, DEMO2 full-length models obtained the highest TM-score (0.575) among all these servers, which was 7.9% higher than that of the second-best server ROSETTA (0.532).

DEMO2 is also compared with the latest version of AlphaFold2 (5) and RoseTTAFold (3) on all CASP14 multi-domain targets. It should be noted that the AlphaFold2 models were regenerated using its latest standalone package rather than from the results reported in CASP14. On average, AlphaFold2 models achieve a considerably higher TM-score ( = 0.842) than that of DEMO2 (0.575), which is probably mainly because of the lower quality of the domain models built by D-I-TASSER. When feeding the domain models predicted by AlphaFold2 into DEMO2, however, DEMO2 obtains a comparable (or slightly higher) TM-score ( = 0.849) to AlphaFold2. If we further input the experimental structure of individual domains, DEMO2 achieved an even higher TM-score ( = 0.891). These results demonstrated that although the overall model quality of DEMO2 relies on the quality of individual domains, DEMO2 has the ability to assemble the domain orientation significantly beyond the state-of-the-art neural-network models when starting from correct domain models. DEMO2 models also obtain a higher TM-score than the RoseTTAFold (0.516), where DEMO2 constructs better models than RoseTTAFold on 70.6% cases. In addition, compared to the deep-learning based end-to-end models which are largely a block box to both developer and users, DEMO2 reports the analogous templates and query-template alignments used to assemble the full-length protein, which can help users better understand where the predictions come from and therefore provide functional insights for further studies on the protein. DEMO2 is also helpful for saving the computational resource for model large-size proteins since it allows the program to split proteins into domains for independent modeling followed by domain assembly. This is very important for the extremely large-size proteins that usually cannot be handled by the end-to-end method.

## WEB SERVER

### Server input

The mandatory input for the DEMO2 server is the individual domain structures in PDB format. Users can click the button ‘Add domain’ (label 1 in Supplementary Figure S9) to add the input box if they have more than two domains needing to be assembled, and the input box also can be removed by clicking ‘Remove domain’ (label 2 in Supplementary Figure S9) if users add a wrong box. In case that users only have sequences, it is suggested that users can submit their sequences of individual domains to the structure prediction servers such as D-I-TASSER (https://zhanggroup.org/D-I-TASSER/), and then upload the domain models into DEMO2 server for full-length structure assembly. A checkbox (label 3 in Supplementary Figure S9) is also provided for inputting the full-chain sequence, which is not required but recommended as the sequence can be used by DEMO2 for dealing with many complex conditions (e.g. missing residues, overlap residues, and wrong domain orders) for domain assembly. Users are also encouraged to provide an email address (but is not required) (label 4 in Supplementary Figure S9) to receive the results when the job is completed. The server will automatically send an email notification containing the link to the results page. Users also can optionally provide a name (label 5 in Supplementary Figure S9) of the target protein. Otherwise, the protein will be named ‘query protein’. The server also provides three advanced options to allow users to design their experiments: (1) provide multiple full-length templates in PDB format to guide the domain assembly; (2) either keep all templates or exclude homologous templates from the library for the domain assembly; (3) upload experiment data including cross-linking and cryo-EM density map data to assist the domain assembly and refinement. All files should be prepared using the format that can be recognized by the server. Details can be found in the instructions by clicking ‘explanation’ for each advanced option. Users can click ‘Run DEMO’ (label 6 in Supplementary Figure S9) to submit the job when all inputs are completed.

### Sever output

Once the job is successfully submitted, the browser will be directed to a new page showing confirmation of the number of domains, the sequence length of the full-chain target, and an estimated time to complete the job (Supplementary Figure S10). Users are recommended to bookmark this link if they do not provide the email address. The results will be displayed on this page when the job is finished. Generally, a medium-size protein (∼300–500 residues) requires 3–8 h for the DEMO2 server. But the actual processing time may be longer if many jobs are pending in the queue.

**Figure 4.**
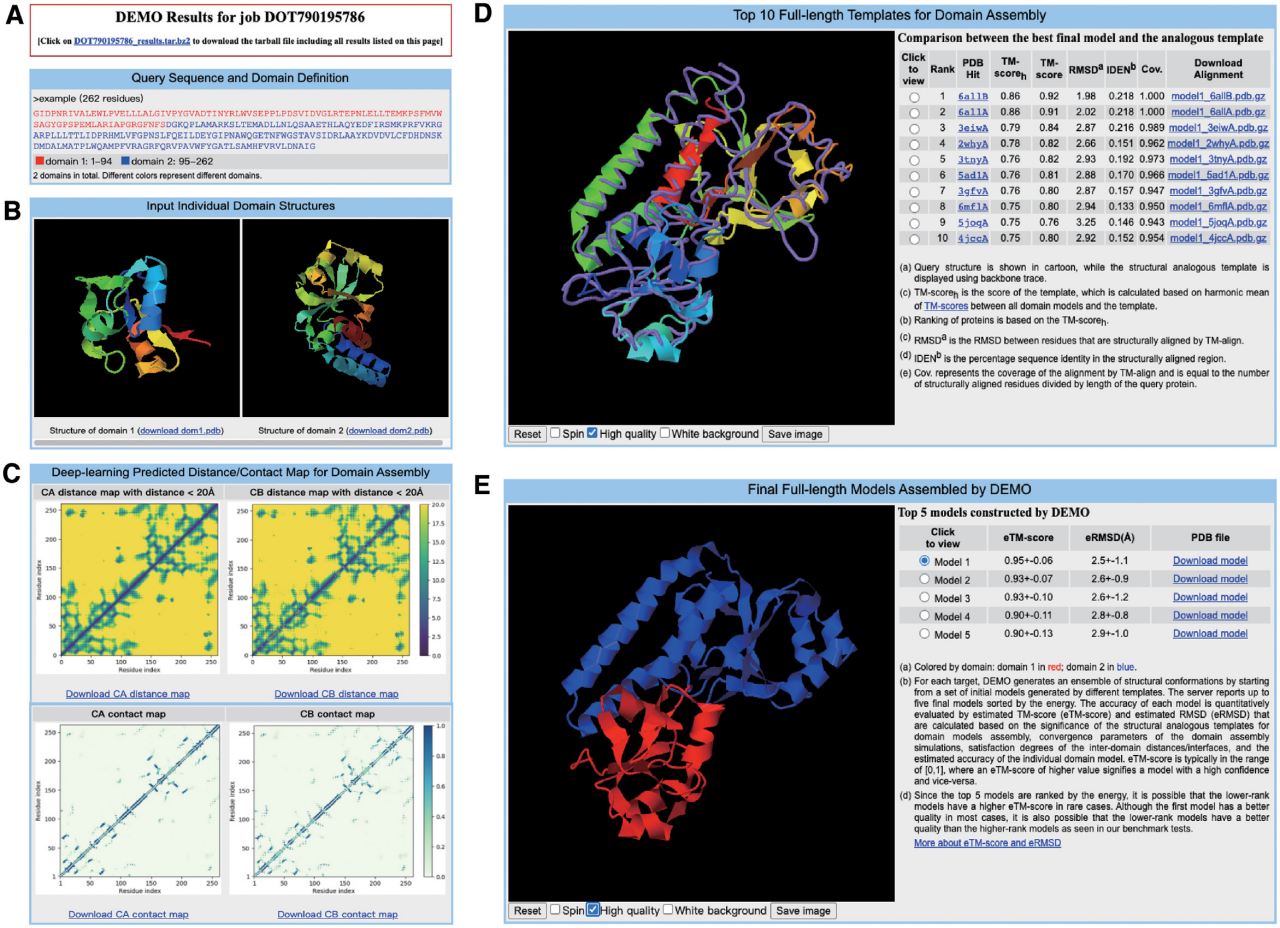
Example of the DEMO2 results page. (**A**) Title of the results page, link to download all results, FASTA sequence, and domain boundaries of the target. (**B**) The user provided domain models for the assembly. (**C**) Predicted residue-residue distance maps and contact maps for domain model assembly. (**D**) The top ten analogous templates identified by the analogous structural alignment. (**E**) Top five final full-length models assemble by the server and the estimated accuracy of the model, where different domains are represented by different colors.

The results page consists of 5 sections, including the full-length sequence and domain boundaries, user submitted domain models, the deep-learning predicted residue-residue distance and contact maps, the top full-length structurally analogous templates, and the top final full-length models assembled by DEMO2. Figure 4 shows an example protein from the *periplasmic ferric siderophore binding* (PDBID: 1efdN), where the model is assembled by selecting the option of ‘remove templates from protein sharing > 30% sequence identity with target’. The result page of the example is available at https://zhanggroup.org/DEMO/example/.

As shown in Figure 4A, the first section shows the job ID of the target and a link to download the tarball file which contains all results reported on the page. The full-length amino acid sequence submitted by the user or translated from the user input domain models is displayed below the title, where the sequence of each domain is represented by different colors. The illustration of the color and the sequence range of each domain are given below the sequence. For example, ‘1–94’ indicates the first domain consists of residues from 1 to 94.

The second section (Figure 4B) shows the 3D model of each domain submitted by the user in an independent JSmol applet (26). Users can rotate and zoom the model by dragging the mouse on the image. The model can also be downloaded by clicking on the ‘download domX.pdb’ link below the image of each domain model.

As shown in Figure 4C, the third section presents the distance and contact maps predicted by DeepPotential. The first and second columns show the inter-residue distance maps with ${C_\alpha }$ and ${C_\beta }$ distances < 20Å, respectively. The third and fourth columns are the inter-residue ${C_\alpha }$ and ${C_\beta }$ contact maps, respectively. Users can click the link labeled ‘Download XXX map’ below each figure to download the corresponding map.

The fourth section (Figure 4D) summarizes the top 10 structurally analogous templates identified from the multi-domain protein library by TM-align using domain models as probe. In the left panel of the JSmol applet, the DEMO2 assembled full-length model is superposed to each template where the DEMO2 model is shown in the cartoon and the template is displayed using backbone trace. The right panel lists the information of all templates, where the proteins are ranked according to the TM-score_h_ reported in the fourth column of the table. The subsequent three columns of the table show the TM-score, RMSD, and sequence identity between the DEMO2 assembled model and the templates. The eighth column is the coverage rate of the aligned regions determined by TM-align. These parameters indicate the conservation of spatial motifs in the model and the structurally analogous proteins. Users can download the PDB file with the DEMO2 model structurally aligned to the corresponding template by clicking the link in the last column of ‘Download Alignment’.

The last section (Figure 4E) presents the top five full-length models assembled by DEMO2. The 3D model of the protein is displayed in the JSmol applet of the left panel, where different colors indicate different domains, and the color is consistent with that illustrated in the first section. The table on the right panel summarizes the five models. Users can click the circle in the first column to display the corresponding model in the JSmol applet. The second column is the estimated TM-score (shown as ‘eTM-score’) for each full-length model. Similar to the standard TM-score, the eTM-score ranges from 0 to 1, where a higher score reflects a model of better quality, and models with eTM-score > 0.5 generally have a correct global fold. The subsequent column reports the estimated RMSD (as ‘eRMSD’) to the native structure. It should be noted that although the first model has a better quality in most cases, it is also possible that the lower-rank models may have a better quality than the higher-rank models as observed in our benchmark tests (27). Users can click on the ‘Download model’ link given in the last column of ‘PDB file’ to download the corresponding model. In addition, users can also view more information about the eTM-score and eRMSD by clicking the link labeled ‘More about eTM-score’.

## CONCLUSION

This work presents a significantly extended server, DEMO2, which integrates analogous template alignments with deep-learning techniques for high-accuracy multi-domain protein structure assembly. Instead of constructing the full-length model by iteratively assembling pair-wise consecutive domain models in the previous DEMO, DEMO2 extended the pipeline to build the full-length model by simultaneously modeling the optimal positions and orientation of all domains through an L-BFGS simulation. Accordingly, the global analogous templates that cover multiple domains are identified in DEMO2 to guide the initial model construction and the assembly simulation. Meanwhile, the inter-domain spatial restraints predicted by the deep residual convolutional networks were coupled with the inter-domain distance profiles collected from the analogous multi-domain templates, and physics-based steric potentials to guide the L-BFGS simulation. In addition, a new confidence score system is introduced to DEMO2 to facilitate the use and interpretation of the assembled models.

DEMO2 was tested on a comprehensive benchmark set of 461 non-homologous proteins containing various numbers and types of domain structures. The results showed that DEMO2 assembled more accurate full-length models with an average TM-score 12.7% and 36.5% higher than DEMO and the linker-based domain assembly method AIDA, respectively. If we compare the rTM-score which counts for the inter-domain orientations more specifically (Eq. S16), the improvement of DEMO2 will be 26.3% and 108.7% over DEMO and AIDA, respectively. DEMO2 was also compared with two widely used deep-learning structure modeling methods, DMPfold and trRosetta. DEMO2 correctly predicted the full-length structure with a TM-score > 0.5 in 84.2% cases, which is 44.2% and 19.0% higher than the full-length models directly modelled by DMPfold and trRosetta, respectively. DEMO2 was also used to assemble structures of all multi-domain targets for ‘Zhang-Server’ in CASP14, which achieved the best performance on the protein modeling among all server groups in the experiment.

To better guide the user to use the method through the online server, we optimized the server interface which includes (i) updating the option for cryo-EM and cross-linking data assisted modeling, (ii) rearranging the result page by adding the superposition of the assembled model, analogous templates, and the accuracy estimated system for the final model. The result page now includes five sections to display the query sequence and domain boundaries, the individual domain models, the residue-residue distance and contact maps, the analogous templates, and the final full-length models.

Despite the progress, the DEMO2 server could be further improved in several aspects. First, the current inter-domain restraints are predicted by DeepPotential, which is developed for the residue-residue restraints prediction for the full-length protein. A specific deep-learning network for predicting the inter-domain restraints should improve the performance. Second, the test of AlphaFold2 has shown the ability to predict the global structure of multi-domain proteins. Although the accuracy of multi-domain models is still considerably worse than that of the individual domains (6), it could be used as new restraints, complementary to the analogous template and DeepPotential restraints, to guide the DEMO2 domain structure assembly. Third, the current DEMO2 server keeps the domain models rigid in the L-BFGS simulations and may limit the range of motion as the predicted domain models often have local errors. The flexible domain assembly combined with specific restraints predicted by deep-learning networks may provide the potential to improve the performance. Finally, the correct inter-domain orientations may be inferred from the protein-protein complex structure library as many multi-domain proteins are evolved from the protein chain fusion/fission (28). Efforts along these lines will continue to improve DEMO as a robust server for multi-domain structure assembly.

## DATA AVAILABILITY

The webserver, standalone package, and dataset are available at https://zhanggroup.org/DEMO/.

## Supplementary Material

gkac340_Supplemental_File
