# Supplementary material for: DEMO2: Assemble multi-domain protein structures by coupling analogous template alignments with deep-learning inter-domain restraint prediction
Source: Nucleic Acids Res. 2022 May 10;50(W1):W235–45. doi: 10.1093/nar/gkac340 (PMC9252800; doi:10.1093/nar/gkac340)
Supplement: gkac340_Supplemental_File [file gkac340_supplemental_file.pdf]

Supplementary Information

Table of Contents

Supplementary Texts

- **Text S1.** Initial full-length model generation by sliding-window based alignment
- **Text S2.** Hybrid energy function for domain assembly
- **Text S3.** Full-length decoys generation using rotation angles and translation vectors
- **Text S4.** Accuracy estimation for DEMO2 model
- **Text S5.** RMSD, TM-score and rTM-score

Supplementary Figures

- **Figure S1.** Global and local templates identification
- **Figure S2.** Sliding-window procedure for domain-template alignment search and initial model construction
- **Figure S3**. Relationship between the eTM-score/eRMSD and the actual TM-score/RMSD to the native
- **Figure S4.** Example of continuous and discontinuous domain
- **Figure S5.** Comparison of DEMO2 with DMPfold and trRosetta
- **Figure S6.** Comparison of DEMO2 with DMPfold and trRosetta on the 162 cases
- **Figure S7.** TM-score comparison of all the individual domain models generated by different methods
- **Figure S8.** Violin plot using the TM-score of models by the top servers of CASP14 for multi-domain targets
- **Figure S9.** Main input page of the DEMO2 server
- **Figure S10.** Example of the job confirmation page of the DEMO2 server

Supplementary Tables

Table S1. Results of full-length models generated by different methods for different categories

Supplementary References

Supplementary Texts

Text S1. Initial full-length model generation by sliding-window based alignment

The initial full-length conformations are built based on the top 10 global templates and local templates selected according to TM-score$_h$. Since the domain alignments are performed separately, the aligned regions of domains may be far away from each other. In this case, a sliding-window based procedure is employed to recreate domain alignments so that neighboring domains have the initial structure constructed from the neighboring regions of the template. Take a protein with 2 domains shown in **Figure S2** as the example, the N-terminal domain of the query is first superposed at the N-terminal of the template, where C-terminal domain is superposed at all the right-hand positions of the N-terminal domain along the template sequence, but with a maximum gap of 10 residues from N-terminal domain. Next, the superposition of N-terminal domain is shifted by one residue to the C-terminal of the template and redo the C-terminal superpositions. This procedure is repeated with the N-terminal domain sliding through all positions along the templates, where C-terminal domain is always on the right hand of the N-terminal domains. To save time, the superposition is initially performed by Kabsch RMSD rotation matrix (1) on all the positions. The top-10 alignment positions with the lowest average RMSD are selected, whose superpositions are then regenerated by the TM-score rotation matrix (2). The alignment with the highest average TM-score of the N/C-domains among all the positions is finally selected for initial model construction. Here, structural superposition without gap (instead of structural alignment with gap) is performed for each comparison of query domain and template structures. The two ending terminals of 20 residues were skipped during domain sliding to further save time.

Text S2. Hybrid energy function for DEMO2 domain structure assembly

The energy function for domain assembly of DEMO2 is a sum of the ten terms:

$$E = \sum_{m=1}^{N_{\text{dom}}} \sum_{n=1}^{N_{\text{dom}}} \Big(w_1 E_{dt}(m,n) + w_2 E_{ori}(m,n) + w_3 E_{it}(m,n) + w_4 E_{EHB}(m,n) + w_5 E_{cl}(m,n) + w_6 E_{ct}(m,n) + w_7 E_{dp}(m,n) + w_8 E_{db}(m,n) \Big) + w_9 E_{tr} + w_{10} E_{rg} \quad \text{(S1)}$$

where *m* and *n* are domain index, and N_{dom} is the total number of domains.

The first term is the *inter-domain* C_β *distance map*:

$$E_{\text{dt}}(m,n) = -\sum_{i=1}^{L_m} \sum_{j=1}^{L_n} \log\left(P\left(i,j,k(d_{ij})\right) + \varepsilon \right) \quad \text{(S2)}$$

where L_m and L_n represent the sequence length of the *m*-th and *n*-th domain, respectively. d_{ij} is the distance between the *i*-th C_β (C_α for Glycine) atom in the *m*-th domain and *j*-th C_β atom in the *n*-th domain, $P\left(i,j,k(d_{ij})\right)$ is the predicted probability of the distance d_{ij} located in the *k*-th distance bin, and $\varepsilon = 1E-4$ is the pseudo count to offset low-probability bins. In the calculation, we only consider atom pairs with probability peak located in [2Å, 20Å], and these atom pairs with predicted probabilities >0.5 in the last bin [>20 Å], which represents a low prediction confidence in [2Å, 20Å], are excluded.

The second term is the *inter-domain orientations*:

$$E_{\text{ori}}(m,n) = -\sum_{i=1}^{L_m} \sum_{j=1}^{L_n} \log\left(P\left(i,j,k(O_{ij})\right) + \varepsilon \right) \quad \text{(S3)}$$

where O_{ij} represents the inter-residue θ, ω, or φ angles defined in Ref. (3), $P\left(i,j,k(O_{ij})\right)$ is the predicted probability of the angle O_{ij} located in the *k*-th angle bin.

The third term is the *domain-domain interface contact energy*:

$$E_{it}(m,n) = \sum_{i=1}^{L_m} \sum_{j=1}^{L_n} \begin{cases} -U_{ij}, & \text{if } d_{ij} < 18\text{Å} \\ -\frac{1}{2} U_{ij} \left[1 - \sin\left(\frac{d_{ij} - 19}{2} \pi \right) \right], & \text{if } 18\text{Å} \le d_{ij} \le 20\text{Å} \\ \frac{1}{2} U_{ij} \left[1 - \sin\left(\frac{d_{ij} - 50}{60} \pi \right) \right], & \text{if } 20\text{Å} < d_{ij} \le 80\text{Å} \\ U_{ij}, & \text{otherwise} \end{cases} \quad \text{(S4)}$$

where U_{ij} is the confidence score of the *i*-th residue and *j*-th residue with the Cα distance <18 Å. A similar potential is also used to count for cross-link restraints when they are available, where U_{ij} is set to 1 with the distance cutoffs taken directly from the user-input cross-link data.

The fourth term is the *hydrogen bond restraints*. The predicted probability distribution of angles is converted into an energy potential with a similar from as the distance energy, where the potential is described as follows:

$$E_{SHB}(m,n) = \sum_{i=2}^{L_m-2}\sum_{j>1}^{L_n-1} E_{SHB}^{AA}(\theta_{ij}^{AA}) + \sum_{i=2}^{L_m-2}\sum_{j>1}^{L_n-1} E_{SHB}^{BB}(\theta_{ij}^{BB}) + \sum_{i=2}^{L_m-2}\sum_{j>1}^{L_n-1} E_{SHB}^{CC}(\theta_{ij}^{CC}) \quad \text{(S5)}$$

$$E_{SHB}^{AA/BB/CC}(\theta_{ij}^{AA/BB/CC}) = -\log\left(\frac{P_{ij}(\theta_{ij}^{AA/BB/CC}) + \varepsilon}{P_{ij}^{N} + \varepsilon}\right) \quad \text{(S6)}$$

where $\theta_{ij}^{AA/BB/CC}$ is the hydrogen angle between residue pair *i* and *j*, i.e. the angle between vector $\overrightarrow{A_i}/\overrightarrow{B_i}/\overrightarrow{C_i}$ and $\overrightarrow{A_j}/\overrightarrow{B_j}/\overrightarrow{C_j}$, which follows a probability distribution P_{ij} predicted by DeepPotential, $P_{ij}(\theta_{ij}^{AA/BB/CC})$ is the probability that the angle is located at $\theta_{ij}^{AA/BB/CC}$. The illustration of the hydrogen bond restraints is shown in (4).

The fifth term is designed to eliminate *steric clashes* between domains, i.e.,

$$E_{cl}(m,n) = \sum_{i=1}^{L_m}\sum_{j=1}^{L_n}\begin{cases}\frac{1}{d_{ij}}, & if\ d_{ij} < d_{cut}\\ 0, & otherwise\end{cases} \quad \text{(S7)}$$

where $d_{\text{cut}} = 3.75$ Å is set as the clash distance cutoff.

The sixth term is the *generic domain-domain contact energy* computed by:

$$E_{ct}(m,n) = \sum_{i=1}^{L_m}\sum_{j=1}^{L_n}\begin{cases}-u_{ij}, & \text{if } d_{ij} < 8\text{Å}\\ -\frac{1}{2}u_{ij}\left[1-\sin\left(\frac{d_{ij}-9}{2}\pi\right)\right], & \text{if } 8\text{Å} \le d_{ij} \le 10\text{Å}\\ \frac{1}{2}u_{ij}\left[1-\sin\left(\frac{d_{ij}-45}{70}\pi\right)\right], & \text{if } 10\text{Å} < d_{ij} \le 80\text{Å}\\ u_{ij}, & \text{otherwise}\end{cases} \quad \text{(S8)}$$

where the scale parameter u_{ij} depends on the hydrophobic and hydrophilic features of the residue pairs. $u_{ij} = 0.1$, if both of the residues are hydrophobic (ALA, CYS, VAL, ILE, PRO, MET, LEU, PHE, TYR, TRP); $u_{ij} = 0.01$, if the two residues are hydrophilic (SER, THR, ASP, ASN, LYS, GLU, GLN, ARG, HIS); or $u_{ij} = 0.05$, otherwise. This energy item is used to control the inter-domain distance, which will push the two domains together if they are two far away each other.

The seventh term is the *domain-domain distance profile* deduced from the templates identified by TM-align, which is calculated by:

$$E_{dp}(m,n) = -\sum_{i=1}^{L_m}\sum_{j=1}^{L_n}\frac{1}{T_{ij}}\sum_{t=1}^{T_{ij}}\frac{1}{|d_{ij} - D_{ij}^{t}|} \quad \text{(S9)}$$

For a residue pair (*i* and *j*, with *i* from N-terminal domain and *j* from C-terminal domain), T_{ij} is the number of templates that satisfy the following two conditions: (1) the template has both residue *i* and *j* aligned by TM-align; (2) $0.6|i-j| < |a_i - a_j| < 1.5|i-j|$, where a_i and a_j are the indexes of the aligned residues of *i* and *j* on the template. D_{ij}^{t} is the C_α distance between the residue a_i and a_j in the *t*-th template.

The eighth term is the *domain boundary energy* is defined as

$$E_{db}(m,n) = (b_{mn} - b_0)^2 \quad \text{(S10)}$$

where b_{mn} is the $C\alpha$ distance between two consecutive domains, and $b_0 = 3.8$ Å is the standard length of $C\alpha$-$C\alpha$ bond.

The nineth term is the *local domain distance restraint*:

$$E_{tr} = \frac{1}{L}\sum_{i=1}^{L} d\,(S_i, S'_i) \quad \text{(S11)}$$

where $d(S_i, S'_i)$ represents the distance between the *i*-th C_α atom (S_i) and its corresponding atom S'_i in the initial structure generated in the template superposition process, and L is the length of the protein. This term is to prevent the assembly deviating too much from the orientation obtained from the template.

The last term is *radius of gyration restraint*, defined as

$$E_{\text{rg}} = \begin{cases}(R_{\max} - R_{\text{decoy}})^2, & \text{if } R_{\text{decoy}} > R_{\max}\\ (R_{\text{decoy}} - R_{\min})^2, & \text{if } R_{\min} < R_{\text{decoy}}\\ 0, & \text{otherwise}\end{cases} \quad \text{(S12)}$$

where R_{decoy} is the radius of gyration of the decoy structure, $R_{\max}$ and $R_{\min}$ are the maximum and minimum estimated radius of gyration, respectively. $R_{\min} = 2.849L^{0.319}$ (L is the query sequence length) is the statistical minimum radius of gyration based on the known multi-domain protein models in the PDB. $R_{\max} = \max\{R_{\min} + 7.5, 0.55N_{\text{mh}}\}$ is the statistical maximum radius of gyration based on the known multi-domain protein models in the PDB, where N_{mh} is the

number of residues of the longest helix.

The weighting parameters in Eq. (S1) are determined by maximizing the correlation between total energy and RMSD to the native on the structure decoys over a training set of 425 non-redundant proteins through a improved differential evolution algorithm (5,6). This resulted in $w_1 = 5$, $w_2 = 1$, $w_3 = 3$, $w_4 = 1.2$, $w_5 = 0.2$, $w_6 = 1.0$, $w_7 = 0.02$, $w_8 = 0.01$, $w_9 = 0.15$, and $w_{10} = 0.13$ for proteins with the template score (TplScore) <0.85, and $w_1 = 1$, $w_2 = 0.2$, $w_3 = 0.2$, $w_4 = 0.15$, $w_5 = 0.15$, $w_6 = 0.1$, $w_7 = 0.02$, $w_8 = 0.01$, $w_9 = 1.2$, and $w_{10} = 0.12$ for other proteins.

Text S3. Full-length structure decoy generation using rotation angles and translation vectors

According to inter-domain rotation angles $\emptyset$, $\boldsymbol{\theta}$, and $\boldsymbol{\psi}$, the rotation matrix can be calculated by

$$a_{11} = \cos\psi\,\cos\emptyset - \cos\theta\sin\emptyset\sin\psi$$
$$a_{12} = \cos\psi\,\sin\emptyset + \cos\theta\cos\emptyset\sin\psi$$
$$a_{13} = \sin\psi\,\sin\theta$$
$$a_{21} = -\sin\psi\,\cos\emptyset - \cos\theta\sin\emptyset\cos\psi$$
$$a_{22} = -\sin\psi\,\sin\emptyset + \cos\theta\cos\emptyset\cos\psi$$
$$a_{23} = \cos\psi\,\sin\theta$$
$$a_{31} = \sin\theta\sin\emptyset$$
$$a_{32} = -\sin\theta\cos\emptyset$$
$$a_{33} = \cos\theta$$

where a_{ij}, *i*=1,2,3, *j*=1,2,3 indicates the element of the matrix. Based on the inter-domain rotation matrix and translation vector, the position of each atom in the domain can be calculated by

$$x_m = t_1 + x_c + (x_0 - x_c)a_{11} + (y_0 - y_c)a_{12} + (z_0 - z_c)a_{13}$$
$$y_m = t_2 + y_c + (x_0 - x_c)a_{21} + (y_0 - y_c)a_{21} + (z_0 - z_c)a_{33}$$
$$z_m = t_3 + z_c + (x_0 - x_c)a_{31} + (y_0 - y_c)a_{22} + (z_0 - z_c)a_{33}$$

where $(t_1,\ t_2,\ t_3)$ is the translation vector of the domain, $(x_0,\ y_0,\ z_0)$ is the initial position of the *m*-th atom, $(x_m,\ y_m,\ z_m)$ is the new position of the *m*-th atom after the transition, $(x_c,\ y_c,\ z_c)$ is the center point of the domain model. The new full-length structural decoy is generated by calculating the position of each atom in each domain according to the corresponding rotation angles and translation vector.

Text S4. Accuracy estimation for DEMO2 model

The accuracy of the DEMO2 assembled model is mainly evaluated by the estimated TM-score (eTM-score), which is calculated based on the convergence of the domain assembly simulations, the confidence of the full-length templates for domain assembly, the satisfaction rate of the inter-domain distances/contacts, and the estimated accuracy of the *k*th individual domain model, i.e.,

$$\text{eTM-score}(k) = w_1\ln\left(\frac{M(k)}{M_{\text{tot}}}\times\frac{1}{\langle\text{RMSD}\rangle_k}\right) + w_2\ln\left(\frac{1}{10}\sum_{i=1}^{10}\frac{\text{TMScore}_{\text{h}}(i)}{\text{TMscore}_{\text{h0}}}\right) + w_3 w_{\text{neff}}\ln\left(\frac{1}{T}\sum_{t=1}^{T}\left|d_t^{\text{pre}} - d_t^{\text{model}}(k)\right|\right)$$
$$+ w_4 w_{\text{neff}}\ln\left(\frac{O(I^{\text{pre}}, I^{\text{model}})_k}{N(I^{\text{pre}})}\right) + w_5\frac{1}{N_{\text{dom}}}\sum_{D=1}^{N_{\text{dom}}}\text{eTM-score}_{\text{dom}}(D) + w_6 \qquad (S13)$$

where M_{tot} is the total number of full-length decoys generated in the domain assembly simulations; $M(k)$ is the number of structure decoys with RMSD <1.5Å to the *k*th reported full-length model; $\langle\text{RMSD}\rangle_k$ is the average RMSD between these decoys and the *k*th reported model. These terms are employed to evaluate the degree of convergence of the domain assembly simulations. $\text{TMScore}_{\text{h}}(i)$ is the template score (i.e., the harmonic mean of the TM-score between the domain model and the DEMO template) of the *i*th full-length template utilized for the initial full-length model construction, and $\text{TMscore}_{\text{h0}}$ (=0.85) is the cutoff of TplScore used to distinguish good templates from bad templates. T is the number of predicted inter-domain distances used to guide the domain assembly; d_t^{pre} and $d_t^{\text{model}}(k)$ are the distances of the *t*th residue pair in the predicted distance map and the reported model, respectively. These terms are applied to assess how closely the distances in the reported model match the predicted distances by DeepPotential. The fourth term accounts for the domain-domain interface satisfaction rate of the predicted interface map in the reported model, where $N(I^{\text{pre}})$ is the number of predicted domain-domain interfaces, and $O(I^{\text{pre}}, I^{\text{model}})_k$ is the number of overlapped interfaces between the predicted interface map and the *k*th reported model. N_{dom} is the total number of domains, and $\text{eTM-score}_{\text{dom}}(D)$ is the estimated TM-score of the *D*th domain model by ResQ (7). $w_1 = 0.065$, $w_2 = 0.063$, $w_3 = -0.08$, $w_4 = 0.01$, $w_5 = 0.96$, and $w_6 = 0.1$ are the weighting factors, which are optimized using an improved differential evolution algorithm (6) to minimize the average error between the eTM-score and the real TM-score of the decoys to the native structure on the DEMO training set with 425 non-redundant multi-domain proteins.

The eRMSD is calculated by the same terms in Eq. (S13) but with an additional term $w_7\ln(L)$ (L is the sequence length of the target), where the weighting factors are $w_1 = -1.40$, $w_2 = -2.74$, $w_3 = 4.78$, $w_4 = -1.19$, $w_5 = -16.43$, $w_6 = 0.0$, and $w_7 = 2.66$.

Text S5. RMSD, TM-score and rTM-score

The most widely used metric for assessing the accuracy of protein structure models is the root mean squared deviation

(RMSD) defined by

$$\text{RMSD} = \min\left[\sqrt{\frac{1}{L}\sum_{i=1}^{L} d_i^2}\right] \quad (S14)$$

where L is the length of the target protein or the number of compared residue pairs, d_i is the distance between the ith pair of compared residues in the model and native structures, and 'min' indicates the rotation matrix to minimize the root mean squared deviation of the two structures (8). Because Eq. (S14) treats the distance error (d_i) with equal weight over all residue pairs, a large local error on a few residue pairs (such as those in the loop or tail regions) can result in a quite large RMSD, even though the global fold of the model is correct. This renders the RMSD value more sensitive to the local error than to the global fold of the assessed model.

TM-score (9) is a metric for evaluating the topological similarity between protein structures, which can be calculated by

$$\text{TM-score} = \max\left[\frac{1}{L_{target}}\sum_{i=1}^{L_{aligned}} \frac{1}{1+\left(\frac{d_i}{d_0(L_{target})}\right)^2}\right] \quad (S15)$$

where L_{target} is the amino acid sequence length of the target protein, $L_{aligned}$ is the length of the aligned residues to the native structure which can be different from L_{target}, e.g., in the case threading alignment with gaps/insertions, $d_0(L_{target}) = 1.24\sqrt[3]{L_{target} - 15} - 1.8$ is a scale to normalize the match difference, and 'max' refers to the optimized value selected from various rotation and translation matrices for structure superposition. The value of TM-score ranges in [0,1], where 1 indicates that the two structures are identical. Stringent statistics showed that TM-score >0.5 corresponds to a similarity with two structures having the same fold defined in SCOP/CATH (10).

Because d_i is put in the denominator of Eq. (S15), TM-score naturally weights smaller distance errors more strongly than larger distance errors. Therefore, TM-score value is more sensitive to the global structural similarity rather than to the local structural errors, compared to RMSD. Another advantage of TM-score is the introduction of the scale $d_0(L_{target}) = 1.24\sqrt[3]{L_{target} - 15} - 1.8$ which makes the magnitude of TM-score length-independent for random structure pairs, while RMSD is a length-dependent metric (9). Due to these reasons, our discussion of modeling results is mainly based on TM-score. Since RMSD is intuitively more familiar to most readers, however, we also list RMSD values, when needed in the manuscript.

Although TM-score is a robust scale for assessing protein fold similarity due to its sensitivity to global fold, it may not appropriately assess the orientation of multi-domain structures for some cases. For a two-domain structure (domain-1 and domain-2) with $L_1 \gg L_2$, for example, the TM-score in Eq. (S15) will be dominated by the tertiary similarity of larger domain, and therefore insensitive to the orientation and quality of the smaller domain. To overcome this issue, we introduce a new score, the reciprocal TM-score (rTM-score), defined by

$$\text{rTM-score} = \frac{N_{dom}}{\frac{1}{\text{TM-score}_1} + \frac{1}{\text{TM-score}_2} + \cdots + \frac{1}{\text{TM-score}_{\text{N}_{\text{dom}}}}} \quad (S16)$$

where TM-score$_k$ is the TM-score for kth domain relative to the native, under the *same* rotation matrix of multi-domain complex structure superposition, and N_{dom} is the number of domains. Please note that rTM-score has a similar form as TM-score$_\text{h}$ defined in Eq (1) but they have different meaning. While in rTM-score the complex model is superposed to the native structure with all domains rotated using the same rotation matrix, the TM-score$_\text{h}$ is the harmonic mean of TM-scores of different domains that are calculated independently. Therefore, the rotation matrixes are different for different domains in the TM-score$_\text{h}$ calculation, which cannot be used to assess inter-domain orientations.

The rTM-score has the value ranging in (0,1), where a rTM-score=1 is achieved if the complex model is identical to the native structure. Compared to TM-score, rTM-score is more sensitive to the domain orientation, as it will approach 0 if only one domain is identical to the native, but the orientation is completely different (i.e., TM-score$_1$~1 and TM-score$_2$~0 for a two-domain protein). In other words, a multi-domain complex model has a high rTM-score only when both the domain tertiary structure and the relative orientation are similar to the native. Here, we consider rTM-score >0.5 as of the correct complex fold. Mathematically, this corresponds to a complex model that has both domains with the similar relative orientation and the similar folds to the native (i.e., TM-score > 0.5) (10).

Supplementary Figures

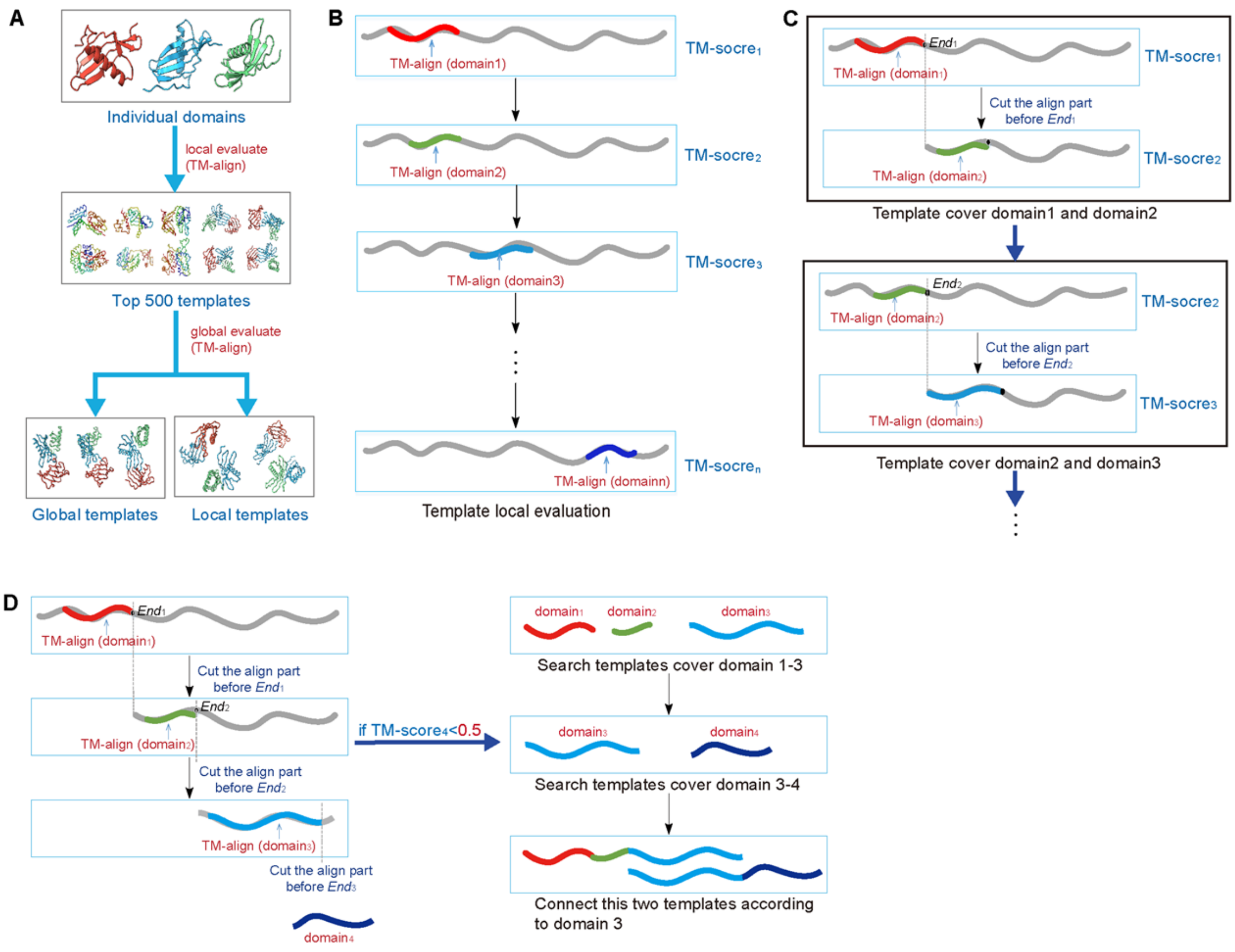

Figure S1. Global and local templates identification. (**A**) Flowchart of the template identification. (**B**) Template local evaluation, where the overlap between the alignments of different domains is allowed. (**C**) Template global evaluation with no overlap allowed in the alignments of different domains. The local template is evaluated by the global evaluation for every two consecutive domains. (**D**) Global template identification, where the fourth domain cannot be covered by the template. Therefore, the templates that can cover domains 1-3 and the templates that can cover domains 3-4 are independently detected from the library. Finally, the initial full-length model is generated by connecting the two templates according to the alignment of domain 3.

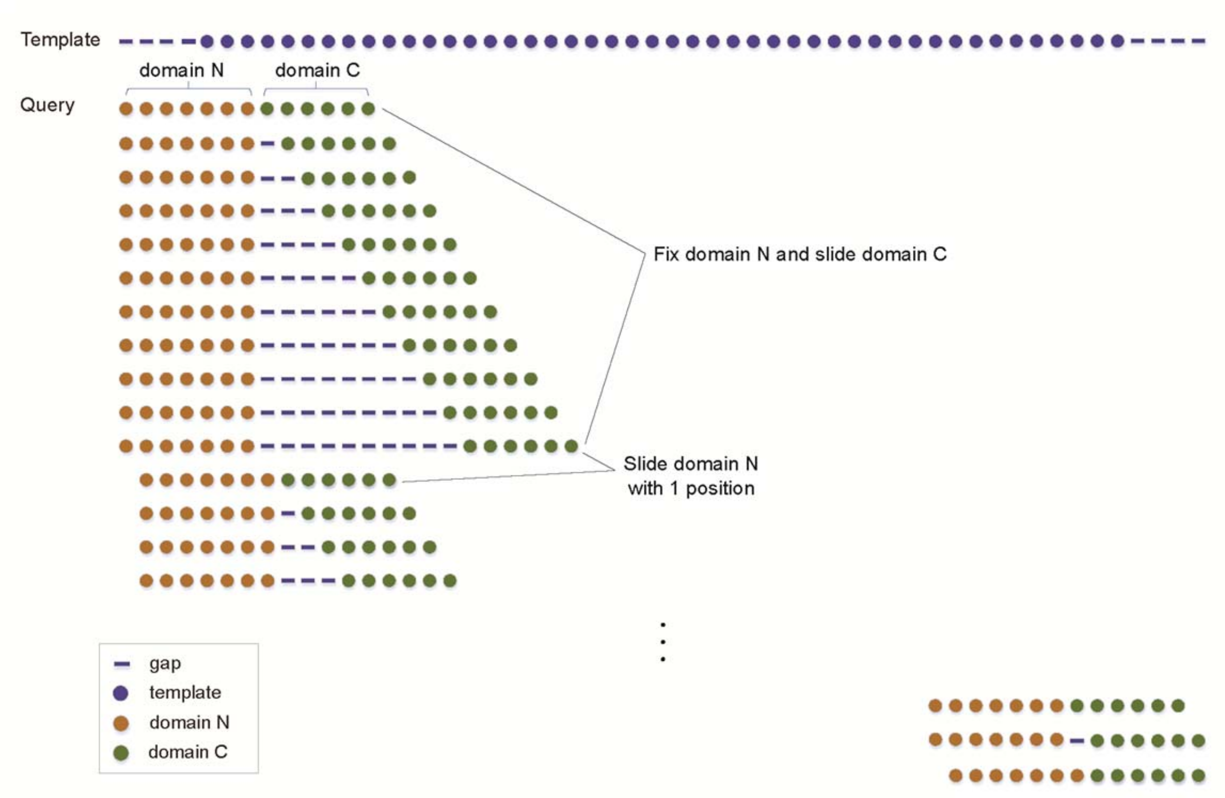

Figure S2. Sliding-window procedure for domain-template alignment search and initial model construction. In this procedure, the N domain is superposed with every position along the template, where at each position, the C domain is allowed to superpose in the remaining regions of the template at a maximum of 10 residues away from the N domain. The alignment with the highest average TM-score is finally selected to construct the initial full-length model for the query sequence.

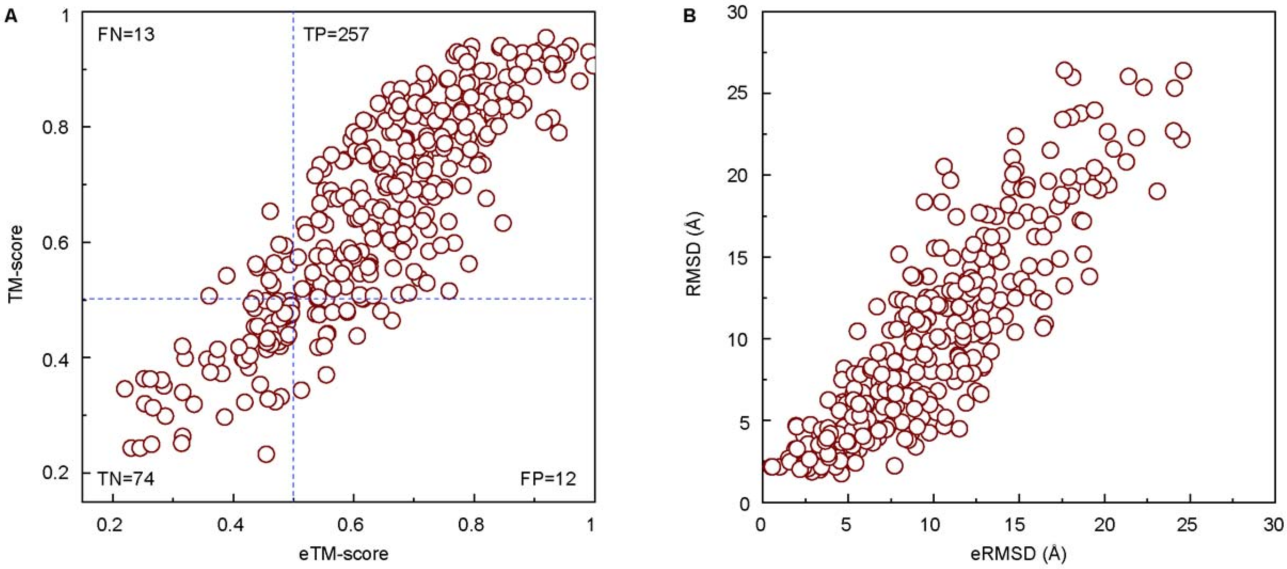

Figure S3. Relationship between the eTM-score/eRMSD and the actual TM-score/RMSD to the native. (**A**) The relationship between the eTM-score and the actual TM-score of the first model assembled by DEMO2, where TP, FP, TN, and FN represent the number of true positive, false positive, true negative, and false negative cases with correct global fold (TM-score > 0.5). (**B**) The relationship between the eRMSD and the actual RMSD of the first model assembled by DEMO2.

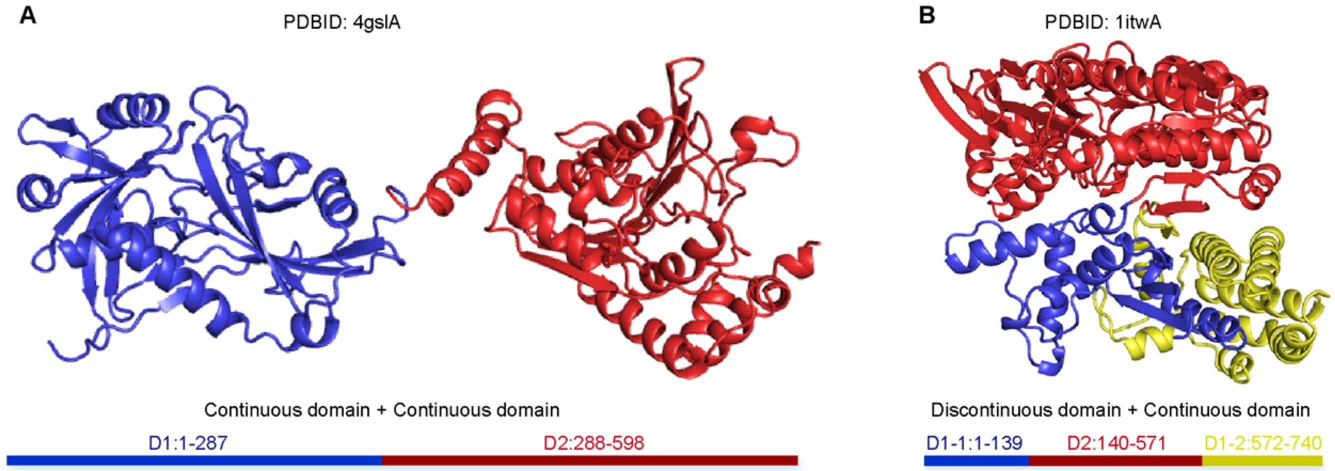

Figure S4. Example of continuous and discontinuous domain. (**A**) A protein (PDBID: 4gslA) contains two continuous domains, where the first domain (blue) ranges from residue 1 to residue 287 and the second domain (red) covers residues from 288 to 598. (**B**) A protein (PDBID: 1itwA) consists of a discontinuous domain and a continuous domain. The first domain is a discontinuous domain which contains two separate segments at the sequence level, where the first segment (blue) ranges from residue 1 to residue 139, and the second segment (yellow) ranges from residue 572 to residue 740. The second domain (red) is a continuous domain inserted between the two segments of the discontinuous domain, and it covers the residues from 140 to 571.

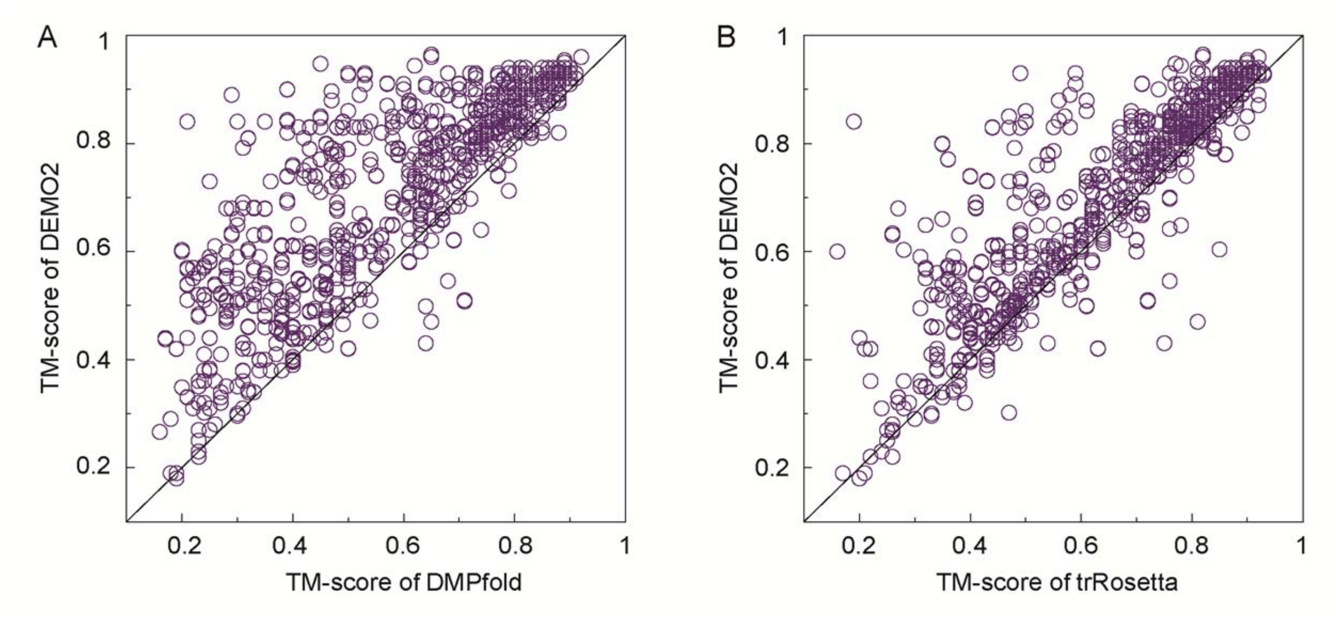

Figure S5. Comparison of DEMO2 with DMPfold and trRosetta. (**A**) Head-to-head TM-score comparison of full-length models generated by DEMO2 and that built by DMPfold. (**B**) Head-to-head TM-score comparison of full-length models generated by DEMO2 and that created by trRosetta.

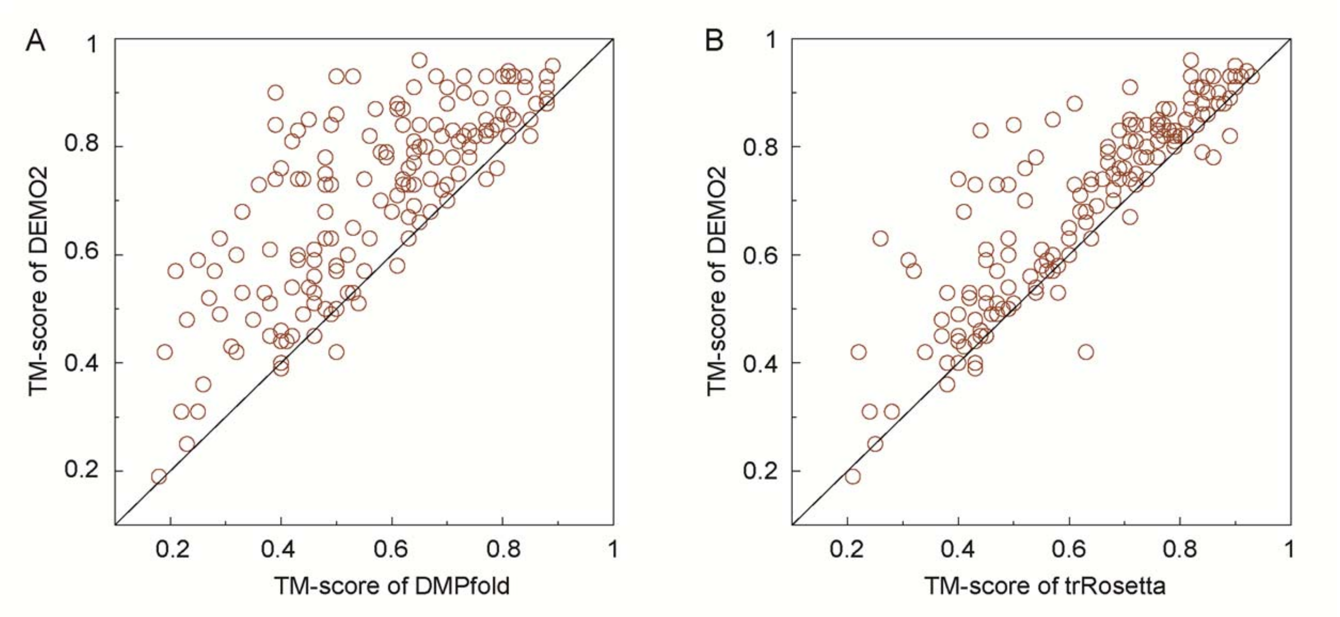

Figure S6. Comparison of DEMO2 with DMPfold and trRosetta on the 162 cases which have no proteins with sequence identity >30% in the DeepPotential training set. (**A**) Head-to-head TM-score comparison of full-length models generated by DEMO2 and that built by DMPfold. (**B**) Head-to-head TM-score comparison of full-length models generated by DEMO2 and that created by trRosetta.

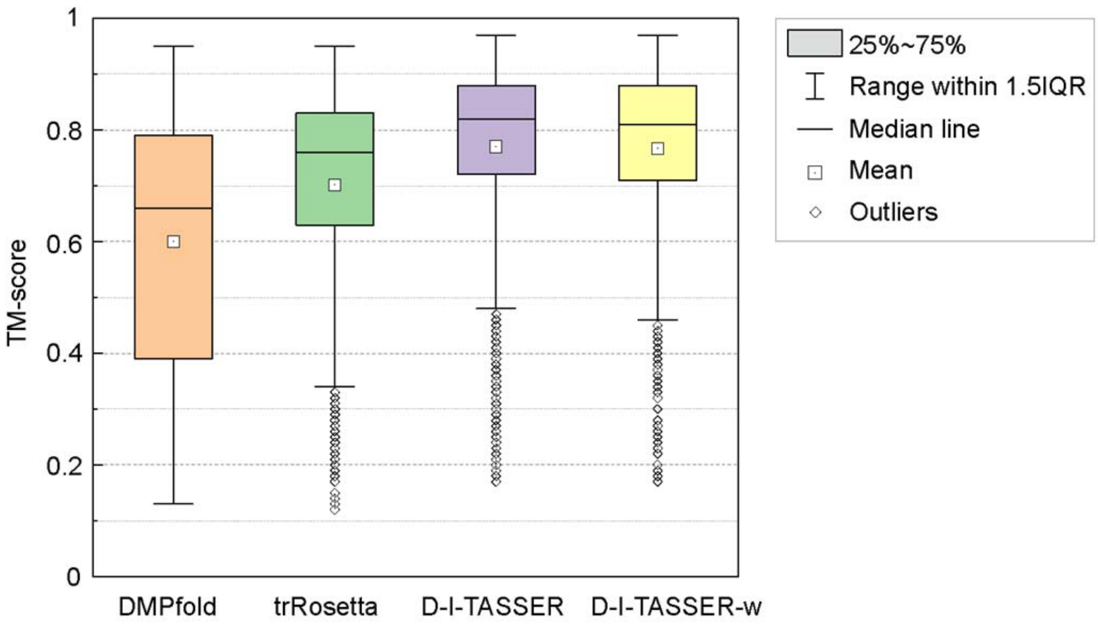

Figure S7. TM-score comparison of all the individual domain models (1202) generated by different methods for all the 461 test proteins, where D-I-TASSER-w indicates D-I-TASSER with both templates with sequence identity >30% and TM-score >0.5 to the query are excluded.

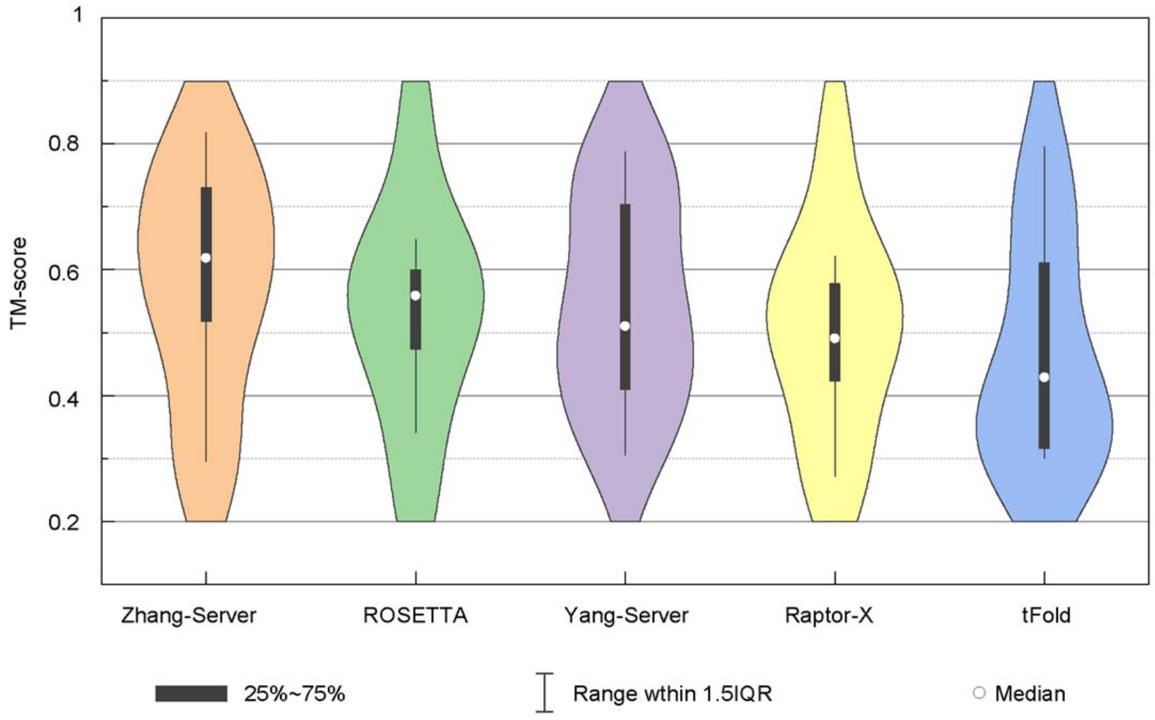

Figure S8. Violin plot using the TM-score of models by the top servers of CASP14 for multi-domain targets. IQR means the interquartile range of the TM-score. Here, we just show the cases with ≥ 1 template-free modeling (FM) or template-free modeling/template-based modeling (FM/TBM) domain since they are usually difficult for modeling.

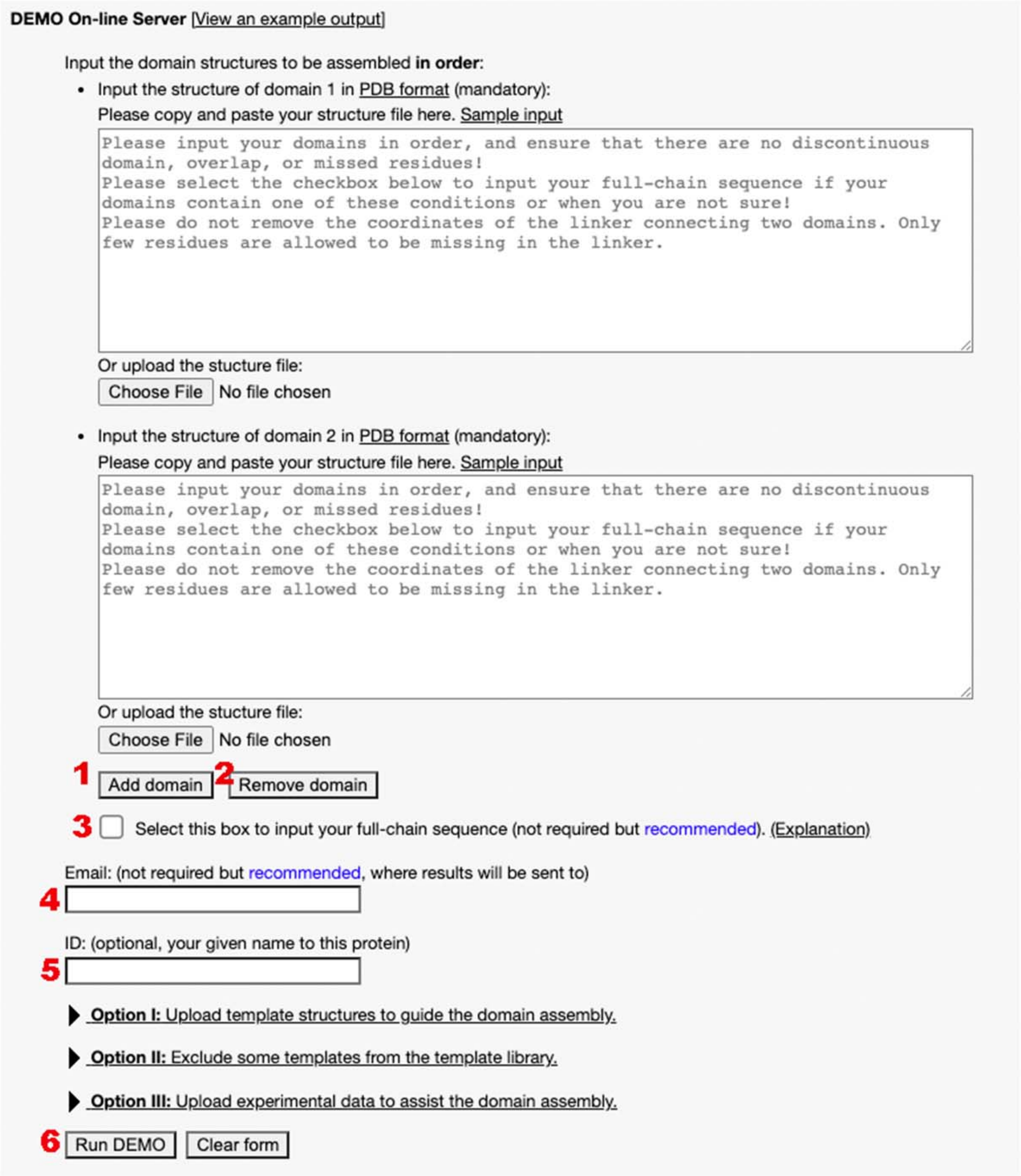

Figure S9. Main input page of the DEMO2 server.

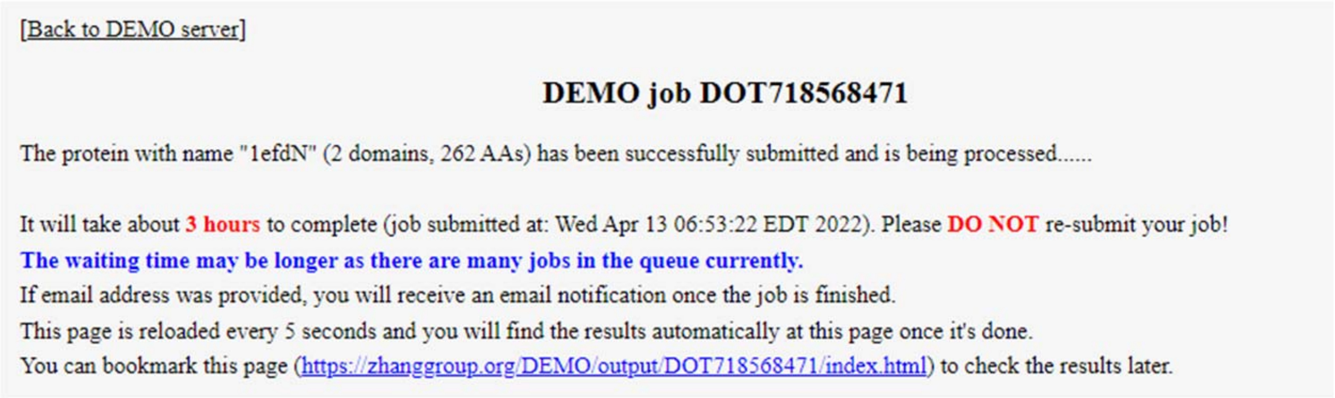

Figure S10. Example of the job confirmation page of the DEMO2 server. The example is a protein from the *periplasmic ferric siderophore binding* (PDBID: 1efdN), which contains two domains with total sequence length = 262.

Supplementary Tables

Table S1. Results of full-length models generated by different methods for different categories. Bold font highlights the best results from each category.

	Category	Method	TM-score	rTM-score	RMSD(Å)
Continuous domain	2dom (N = 155)	AIDA	0.57	0.27	13.6
		DEMO	0.64	0.39	11.0
		DMPfold	0.56	0.33	12.8
		trRosetta	0.63	0.42	10.3
		DEMO2	**0.70**	**0.48**	**8.9**
	3dom (N = 65)	AIDA	0.47	0.14	19.0
		DEMO	0.57	0.28	14.1
		DMPfold	0.51	0.23	16.4
		trRosetta	0.57	0.31	13.1
		DEMO2	**0.64**	**0.36**	**11.0**
	m4dom (N = 40)	AIDA	0.37	0.08	25.1
		DEMO	0.44	0.15	21.0
		DMPfold	0.44	0.15	23.4
		trRosetta	0.54	0.23	16.5
		DEMO2	**0.60**	**0.27**	**15.3**
	All (N = 260)	AIDA	0.52	0.20	16.7
		DEMO	0.59	0.32	13.4
		DMPfold	0.53	0.28	15.4
		trRosetta	0.60	0.36	12.0
		DEMO2	**0.67**	**0.42**	**10.4**
Discontinuous domain	2dom (N = 149)	AIDA	0.58	0.28	14.1
		DEMO	0.69	0.50	10.0
		DMPfold	0.63	0.45	11.0
		trRosetta	0.69	0.51	9.7
		DEMO2	**0.75**	**0.60**	**7.4**
	3dom (N = 33)	AIDA	0.49	0.28	15.4
		DEMO	0.69	0.30	12.1
		DMPfold	0.63	0.36	12.6
		trRosetta	0.73	0.46	9.6
		DEMO2	**0.78**	**0.52**	**8.5**
	m4dom (N = 19)	AIDA	0.31	0.17	27.4
		DEMO	0.54	0.27	23.0
		DMPfold	0.58	0.25	20.0
		trRosetta	0.66	0.30	14.6
		DEMO2	**0.70**	**0.34**	**13.0**
	All (N = 201)	AIDA	0.54	0.27	15.5
		DEMO	0.68	0.46	11.6
		DMPfold	0.63	0.42	12.1
		trRosetta	0.69	0.48	10.1
		DEMO2	**0.75**	**0.56**	**8.1**

2dom: protein with 2 domains.
3dom: protein with 3 domains.
m4dom: protein with 4 or more domains.
Discontinuous domain: protein contains ≥ 1 domains which consist of ≥ 2 segments from separate regions of the query sequence.

Supplementary References

1. Kabsch, W. (1978) A discussion of the solution for the best rotation to relate two sets of vectors. *Acta Crystallographica Section A: Crystal Physics, Diffraction, Theoretical General Crystallography*, **34**, 827-828.
2. Zhang, Y. and Skolnick, J. (2004) Scoring function for automated assessment of protein structure template quality. *Proteins*, **57**, 702-710.
3. Yang, J., Anishchenko, I., Park, H., Peng, Z., Ovchinnikov, S. and Baker, D. (2020) Improved protein structure prediction using predicted interresidue orientations. *Proceedings of the National Academy of Sciences*, **117**, 1496-1503.
4. Yang, J., Yan, R., Roy, A., Xu, D., Poisson, J. and Zhang, Y. (2015) The I-TASSER Suite: protein structure and function prediction. *Nature methods*, **12**, 7-8.
5. Zhou, X.G. and Zhang, G.J. (2017) Abstract convex underestimation assisted multistage differential evolution. *IEEE transactions on cybernetics*, **47**, 2730-2741.
6. Zhou, X.G., Peng, C.X., Liu, J., Zhang, Y. and Zhang, G.J. (2020) Underestimation-assisted global-local cooperative differential evolution and the application to protein structure prediction. *IEEE Transactions on Evolutionary Computation*, **24**, 536-550.
7. Yang, J., Wang, Y. and Zhang, Y. (2016) ResQ: an approach to unified estimation of B-factor and residue-specific error in protein structure prediction. *Journal of molecular biology*, **428**, 693-701.
8. Kabsch, W. (1976) A solution for the best rotation to relate two sets of vectors. *Acta Cryst*, **A 32**, 922-923.
9. Zhang, Y. and Skolnick, J. (2004) Scoring function for automated assessment of protein structure template quality. *Proteins*, **57**, 702-710.
10. Xu, J. and Zhang, Y. (2010) How significant is a protein structure similarity with TM-score = 0.5? *Bioinformatics (Oxford, England)*, **26**, 889-895.
